# Comparative analysis of reinforcement learning and artificial neural networks for inverter control in improving the performance of grid-connected photovoltaic systems

**DOI:** 10.1038/s41598-025-09507-9

**Published:** 2025-07-08

**Authors:** Saad A. Mohamed Abdelwahab, Hossam Eldin Khairy, Hossam Yousef, Samia Abdafatah, Moayed Mohamed

**Affiliations:** 1 Department of Electrical, Faculty of Technology and Education, Suez University, P.O.Box: 43221, Suez, Egypt; 2 Electrical Technology Department, Faculty of Technology and Education, Helwan University, Helwan, 11795 Egypt; 3 Electrical Department, Faculty of Technology and Education, Sohag University, Sohag, Egypt

**Keywords:** PV, Voltage source control (VSC), MPPT, RL, ANN, Engineering, Electrical and electronic engineering

## Abstract

This research aims to explore the potential applications of artificial intelligence (AI) methods, such as reinforcement learning (RL) and artificial neural networks (ANN), in controlling inverter systems and enhancing the performance of photovoltaic (PV) systems. PV systems are essential for producing sustainable energy, as they improve the reliability and efficiency of renewable power resources by utilizing AI to control inverters. This study examines the application of AI techniques to manage PV systems, given the increasing importance of energy generation through PV systems on a global scale. The goal of the project is to investigate the potential applications of RL algorithms for achieving maximum power point tracking (MPPT) and managing PV system maintenance and operation. According to the results, control of the inverter by RL yields better results than the ANN controller in all cases. Globally, increasing the use of PV systems for energy generation is a top goal to satisfy rising energy demands sustainably. By improving efficiency and dependability, AI control of PV systems helps to meet this challenge and further efforts in environmental sustainability and energy security. In terms of efficiency, reliability, and overall system performance, the research findings demonstrate that RL-based control of inverters outperforms ANN controllers. This comparison highlights how well RL works to control PV systems adaptively and efficiently in various environmental conditions. Total Harmonic Distortion (THD) for both current and voltage is compared and evaluated under ramp and random conditions. The results show that by consistently achieving reduced THD values, the RL controller outperforms the ANN controller in both dynamic and uncertain scenarios. This study reveals that RL exhibits superior adaptability and achieves lower THD compared to ANN, particularly under varying operational conditions. This comparative analysis fills a significant research gap, as comprehensive evaluations of this nature have not been adequately addressed in previous works. These results highlight how RL approaches may increase the dependability and efficiency of PV systems, advancing sustainable energy technology.

## Introduction

As the globe shifts from diesel to renewable energy, the need for clean and green sources of energy grows. Renewable energy, often referred to as clean energy, plays a crucial role in reducing pollution and mitigating the adverse effects of fossil fuel-based energy on global air quality. This shift toward renewable energy sources underscores the pressing need for innovative and advanced solutions in energy technology^1^. PV systems are becoming an increasingly important source of renewable energy as the need for environmentally friendly and sustainable solutions grows. With the increasing development of the solar energy industry and technical breakthroughs, the performance of PV systems must be continuously improved^2^. These systems utilize sunlight to generate energy, significantly reducing carbon emissions and promoting environmental sustainability^3^.

Two methods of artificial intelligence (AI) techniques are used in this paper to control the inverters of PV grid-connected systems. The types of AI are RL and NN. This work proposes the use of AI techniques, specifically RL and ANN, to enhance the control of inverters in PV systems. Furthermore, the paper discusses the benefits of using AI techniques in inverter control, such as improved power point tracking, increased reliability, and getting the best maximum point. Objectives: to use the application of AI techniques, specifically RL, for improving control in the inverter of PV panels connected to the grid. The control techniques employed in inverters have a significant impact on the efficiency and power quality of solar systems. Inverters convert direct current (DC) from PV arrays into alternating current (AC) electricity, making it suitable for grid integration^4^. Inverter control schemes aim to maximize MPPT and energy conversion efficiency, thereby improving overall PV system performance^5,6^.

Despite advancements in PV technology, significant hurdles remain to maximizing the performance and reliability of PV systems. Variations in solar irradiation, temperature changes, shading effects, and system aging are all potential hurdles to the efficiency and production of PV systems^5^. To address these problems, novel techniques and sophisticated technology are required to ensure that PV systems work consistently and optimally^5^.

In this paper, two types of AI techniques were employed for controlling the PV grid-connected system: the first is ANN, and the second is RL. These types are called Machine Learning (ML). ANN is the first and simplest type of all AI techniques; models rely on historical data to predict control actions, providing a degree of accuracy and flexibility in PV system control^6^.

RL is an advanced AI technique inspired by human learning processes. It demonstrates the ability to learn optimal control policies through iterative interactions with the environment, enabling adaptive and efficient operation of PV systems^7^.

To overcome these difficulties and improve the performance of the PV system, the authors are investigating the application of AI tools. AI offers promising methods for enhancing PV system operation and control, utilizing algorithms that can analyze vast amounts of data and make real-time decisions to improve system efficiency^8^. One of the most promising AI approaches for PV systems is ML, which entails creating algorithms that can learn from data and adjust their behavior in response to patterns and trends^9^.

ML algorithms may find intricate links and improve control techniques in PV systems by evaluating vast volumes of data from sensors, weather stations, and previous performance records. This enables operators to anticipate system performance in various environmental scenarios, make data-driven decisions, and implement proactive maintenance plans to minimize downtime and maximize energy output^10^. Furthermore, improvements in ML algorithms, such as ANN methods, have expanded AI’s potential for optimizing PV system efficiency. With the application of multi-layered NNs, which feature the hallmark of ANN algorithms, complex characteristics may be extracted from data, allowing for more precise control methods and predictions in PV systems. The integration of AI methods, including ML, with PV system control signifies a noteworthy paradigm change in renewable energy technology. Operators can overcome conventional control strategy limitations and achieve improved levels of efficiency, dependability, and sustainability in PV systems by leveraging AI capabilities^11^. The use of AI approaches for controlling and optimizing PV systems has been the subject of several studies and research efforts in recent years. According to this research, AI algorithms may effectively improve MPPT accuracy, energy conversion efficiency, and overall system performance^12^. Moreover, AI is being utilized in PV systems for tasks such as defect detection, predictive maintenance, and component optimization, extending beyond control techniques. Inverters, batteries, and monitoring devices are examples of PV system components whose data can be analyzed by ML algorithms, enabling proactive maintenance interventions and early problem identification^13^. Recent advancements in ANN techniques have shown promise in improving the control and optimization of PV systems. ANN-based controllers utilize neural network (NN) models trained on historical data to predict optimal control actions, offering improved accuracy and flexibility compared to traditional methods^13^.

This paper introduces the significance of PV systems and highlights the critical role of inverters in grid-connected configurations. It emphasizes the necessity of employing advanced control techniques, such as AI, to enhance the efficiency and performance of these systems. Several studies examining AI applications in PV systems have shown significant improvements in control and performance. The objective of this research is to explore the potential of AI methods, particularly ANN, in enhancing PV system performance and optimizing inverter management.

Many past studies have focused on integrating renewable energy systems using AI techniques. In^14^, it was demonstrated how AI techniques are used to control the rotor-side converter in DFIG. This comparison of AI methods reveals that deep learning controllers yield better results than ANN controllers under various conditions. In^15^, a PID Controller with grid-connected HRES using optimization algorithms demonstrates reliable MPPT performance under varying solar irradiance levels. The linear quadratic Gaussian-based PID controller is applied in^16^ to maximize the frequency stability of power systems based on renewable energy. To improve system performance under various environmental conditions, the authors in^17^ proposed the use of an adaptive sine cosine algorithm (ASCA) to optimize the PID controller settings in an HRES. However, conventional control techniques, such as PID controllers, are not as flexible in responding to changing environmental conditions, and therefore cannot fully utilize the potential of PV systems, particularly in grid-connected applications where the optimal power output is essential.

In^18^, an ANN-based MPPT and the GRG algorithm are employed to demonstrate an optimized LCL filter for improved power quality in a grid-connected PV system. Demonstrated that ANN-based controllers can successfully operate grid-connected PV systems with greater MPPT accuracy and energy conversion efficiency. An ANN-based system’s capacity to dynamically optimize system performance in response to external conditions and real-time data may help overcome the limitations of conventional control techniques. Ali et al. developed two different AI-based MPPT systems for PV units connected to a grid^19^. Their MPPT approaches included fuzzy logic, metaheuristic, and ANN techniques. PV efficiency, output power, and tracking speed were all optimized by these systems.

In^20^ verification through experimental in comparison to traditional methods, the authors validate the adaptive PID-ANN for regulating DC link voltage in a PV converter using dSPACE within the ANN training in classic PID control of the DC link for feeding solar energy into the grid, demonstrating improved performance with minimal overshoot and lower total harmonic distortion (THD).

In^21^, a control system for a DC microgrid with renewable energy sources and an energy management system using ANN was introduced. Simulation experiments validate its stability and improved load-generation balance. In^22^, the authors used types of AI’s impact on fuzzy and neuro-fuzzy accuracy and precision in the transmission line to measure fault identification. In^23^, D Gueye et al. trained an AI to optimize the best solution for controlling PV systems in smart grid connection and experimented with this system controller. The authors of^24^ investigate the application of AI strategies for enhancing the performance of inverters in Solar panel systems to achieve the MPPT of PV generation. Additionally, in^25^, the authors explain that energy explores the integration of renewable energy systems with AI algorithms to enhance grid stability and power quality. Several comparative studies have evaluated the performance of ANN-based controllers against traditional control methods in PV systems that are connected to the grid. For instance, in^26^, Moayed Mohamed et al. compared the performance of PID, SMC, and ANN controllers in solar panel systems that are connected to the grid under varying weather conditions, the results showed that the ANN-based controller showed better performance than traditional methods in terms of MPPT accuracy and energy generation efficiency, highlighting the potential benefits of ANN integration in PV system control. Many other studies sought the advantages of ANN-based control in PV systems. In^27^, the authors have sought to improve the efficiency of PV systems through MPPT inverters, with ANN proving advantageous for fast and accurate MPPT, achieving average performance and suitability for changing conditions compared to other intelligent algorithms.

Existing studies have shown promising results with AI-based control of PV systems; however, a significant gap remains in exploring RL techniques specifically for grid-connected applications. RL, as a relatively less explored method compared to ANN controllers, presents a novel avenue for enhancing the performance and dependability of inverter control in PV systems, highlighting the importance of this study in filling this gap and advancing research in this emerging area.

This work aims to bridge this gap by emphasizing the unique advantages of RL and its potential to raise the effectiveness of renewable energy systems. The importance of PV, the technology of solar panels, and the role of inverters in grid-connected PV modules are highlighted at the outset of this study. It emphasizes the need for enhanced AI controller strategies to improve system efficiency and performance. Numerous research studies examining AI approaches in PV systems have shown enhanced performance and efficiency, as documented in the literature study. This work attempts to address a significant gap in the field by exploring RL approaches for grid-connected applications. By utilizing AI agents’ learning capabilities to develop intelligent control schemes that enhance system performance, reduce downtime, and improve energy production, the importance of PV is emphasized. The technology of solar panels and the role of inverters in grid-connected PV modules are highlighted at the outset of this study.

Table 1 provides a summary of previous studies on AI applications and control strategies in renewable energy systems, including system components and methods used. The research features cutting-edge methods, including ANN, FLC, and optimization algorithms, and covers a range of topologies, including microgrids, hybrid systems, and PV systems.

This approach demonstrates how RL achieves superior adaptability and significantly lower THD compared to ANN. Unlike previous studies, this work provides a comprehensive evaluation under dynamic conditions, highlighting the robustness of RL in real-time inverter control for grid-connected PV systems.

An ANN controller and an RL controller are employed in a grid-connected photovoltaic system, the subject of a notable case study. This case study illustrates how AI can be leveraged to address real-world challenges in renewable energy management by showcasing the effective integration of AI-driven strategies to optimize system performance and flexibility.

**Table 1:** Comparative summary of previous studies on AI-Based control in renewable energy systems.

Ref.	Year	System components	Technique type	Application focus
^1^	2022	Multi-source generators (thermal, hydro, gas)	Equilibrium Optimizer + Fuzzy Logic	Load frequency control
^2^	2023	PV array, ANN-based MPPT controller, LCL filter	ANN + GRG optimization	MPPT in grid-connected PV
^3^	2020	PV systems with inverters and storage	Comparative analysis	Controller performance evaluation
^4^	2021	Hybrid PV-wind system with battery	Optimization algorithms	Economic/environmental optimization
^5^	2022	PV array, microgrid, grid interface	Levenberg-Marquardt ANN	Intelligent inverter control
^6^	2021	AI-based EMS, wireless sensor networks	AI + IoT	Energy management
^7^	2020	PV panels, inverter, synchronization units	MPPT + sync control	Grid-connected operation
^8^	2022	PV + battery microgrid	ANN, PID, Fuzzy Logic	Multi-controller integration
^9^	2023	Energy and climate modeling systems	AI analytics	Forecasting and planning
^10^	2020	PV standalone system	ANN sizing algorithm	System design and optimization
^11^	2022	Smart microgrid with DG units	AI-based controller	Load sharing & power quality
^12^	2022	DC microgrid with PV & battery	Nonlinear AI control	Distributed energy control
^13^	2021	PV with AI-enhanced inverters	AI control schemes	Grid stability
^14^	2024	DFIG wind turbine system	AI optimization	Rotor current control
^15^	2021	Hybrid PV-wind with PID controller	PID + optimization	MPPT tracking
^16^	2021	Power system with renewables	PID + LQG control	Frequency stability
^17^	2020	Hybrid PV-wind system	Adaptive Sine Cosine Algorithm	PID tuning
^18^	2021	PV system with shading	MPPT algorithm comparison	Partial shading analysis
^19^	2021	Grid-connected PV system	Metaheuristic, Fuzzy Logic, ANN	MPPT optimization
^20^	2017	Flexible actuator system	Fuzzy PID	Intelligent joint control
^21^	2022	DC microgrid with PV and wind	Nonlinear AI controller	Energy control
^22^	2024	High-voltage transmission network	Fuzzy-neuro-fuzzy	Fault detection
^23^	2022	PV + grid interface + ANN-PID	ANN-PID controller	DC link voltage regulation
^24^	2021	PV system with MPPT	ANN optimization layers	Power extraction efficiency
^25^	2020	Control systems	Deep Q-learning	RL in control applications
^26^	2021	PV-wind system with ANN-SMC	ANN + Sliding Mode Control	Grid regulation
^27^	2018	PV + MPPT	ANN-based MPPT	Fast MPPT tracking
This Study	—	Grid-connected PV system	Reinforcement Learning (RL) and ANN	Comparative MPPT and inverter control under dynamic conditions
Ref.	Year	System Components	Technique Type	Application Focus

This research aims to investigate how RL approaches may be applied to enhance PV system performance. Intelligent control techniques that improve energy generation with PV systems can be developed by leveraging the learning capabilities of AI agents. The primary goal is to advance sustainable energy technologies by increasing energy output, reducing downtime, and enhancing the overall efficiency of PV systems.

## System description

**Fig. 1:**
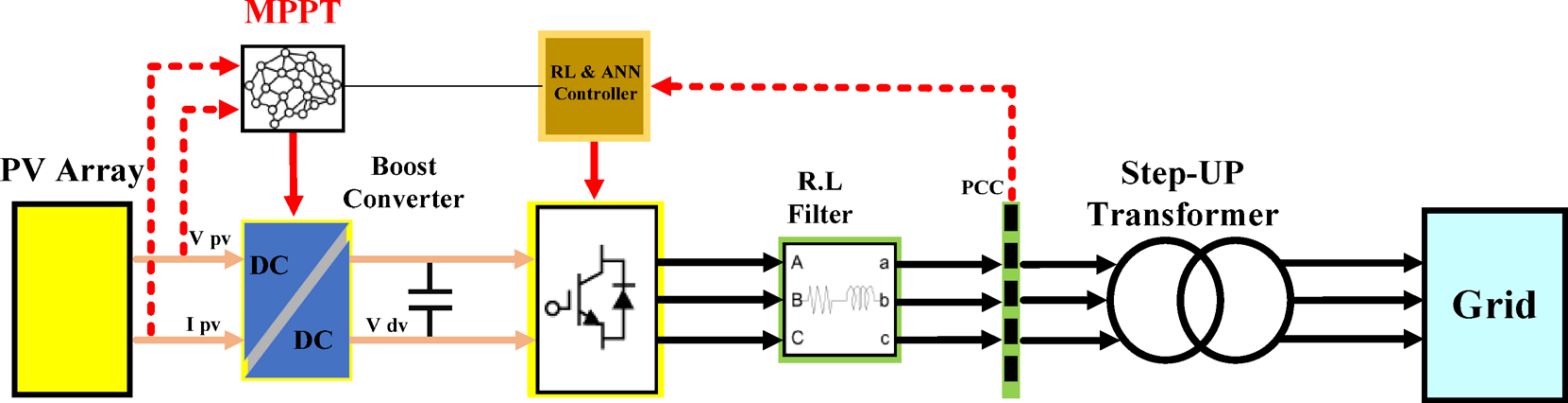
The structure of the PV system for renewable energy generation.

The PV system described in this study, which includes a PV array, boost converter, DC link, three-phase type inverter, controller unit, step-up transformer, and power grid are depicted in Fig. 1. The DC-AC converter is essential to the system by enabling the (DC) from the PV panels to be converted into (AC) to satisfy the utility grid’s demands.

The functionality and effectiveness of the system depend on each component. One kind of DC-DC converter that boosts the voltage generated by the PV panels to the level needed by the DC link or inverter is called a boost converter. This guarantees grid compatibility, balancing the grid’s voltage and frequency with the generated power output. The controller plays a vital role in system management by maximizing the efficiency of the boost converter and the inverter. It controls voltage and frequency, guarantees MPPT, and upholds system stability. To improve the controller’s efficiency, this study presents AI approaches, including RL and ANN. The controller is a crucial component of the proposed PV system, as it can achieve more accurate voltage regulation, an effective MPPT, and enhanced system stability by utilizing these AI techniques.

### PV system

PV panels are primarily used in grid-connected systems to generate electricity for appliances, equipment, commercial air conditioning, and lighting in modern buildings. PV panels could be installed on building rooftops, grounded, and building materials designed into the manufacturing process at the point^28–31^. The MPPT with PV panels in the HRE system uses the Incremental Conductance technique. The root mean square (RMS) voltage, which is supplied by the PV module’s DC voltage (Vdc), is described by the PV output voltage Eq.

1 $$\:{\text{V}}_{\text{r}\text{m}\text{s}}=0.612\:{\text{M}}_{\text{a}}{\text{V}}_{\text{d}\text{c}}$$.

In analyzing and designing PV control systems, the mathematical representation of PV panels is a crucial stage. The following equation represents the current and voltage of the PV cell:

2 $$\:{I}_{c}={I}_{ph}-{I}_{o}\left\{{e}^{\left[\frac{q}{AkT}\left({V}_{c}+{I}_{c}{R}_{s}\right)\right]}-1\right\}$$

3 $$\:{V}_{c}=\frac{AkT}{q}{ln}\left(\frac{{I}_{ph}+{I}_{o}-{I}_{c}}{{I}_{o}}\right)-{I}_{c}{R}_{s}$$

4 $$\:I={I}_{ph}-{I}_{o}\left\{{e}^{\left[\frac{q}{{n}_{s}AKT}\left(V+{n}_{s}I{R}_{s}\right)\right]}-1\right\}$$

5 $$\:V=\frac{{n}_{s}AkT}{q}{ln}\left(\frac{{I}_{ph}+{I}_{o}-I}{{I}_{o}}\right)-{n}_{s}I{R}_{s}$$

Where I_e_ is the output current of the PV cell, V_e_ is the output voltage of the PV cell, I_o_ represents the diode’s saturation current, I_ph_ is the photon-generated current, R_s_ represents the series resistance measured in ohms, The parallel resistance, denoted as R_sh_ and measured in ohms, the ideality factor denoted as A, K represents the Boltzmann constant, g represents the electron charge, and The temperature, denoted as T and measured in Kelvin.

6 $$\:{I}_{ph}=\frac{G}{1000}\left[{I}_{sc}+{k}_{i}\left(T-{T}_{r}\right)\right]$$

7 $$\:{I}_{o}={I}_{or}{\left(\frac{T}{{T}_{r}}\right)}^{3}{e}^{\left[\frac{q{E}_{g}}{AK}\left(\frac{1}{{T}_{r}}-\frac{1}{T}\right)\right]}$$

As demonstrated in (6), the module output power can be easily detected.

8 $$\:\text{P}=\text{V}\text{I}$$.

**Fig. 2:**
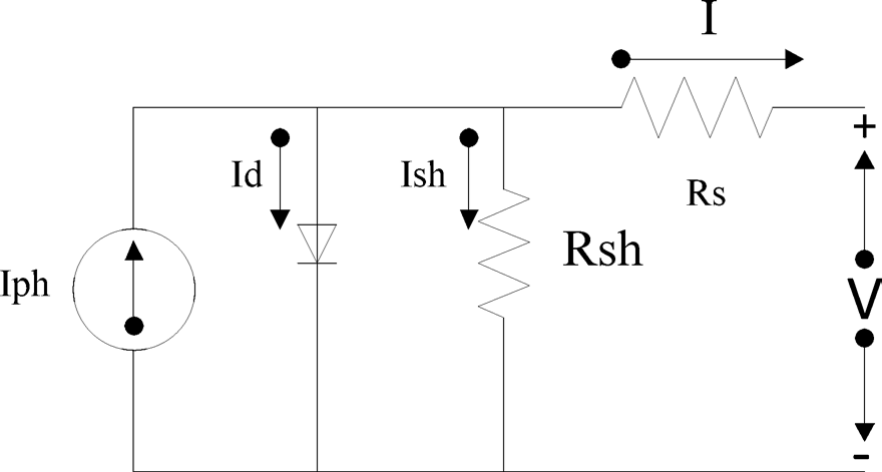
PV cell equivalent circuit.

**Fig. 3:**
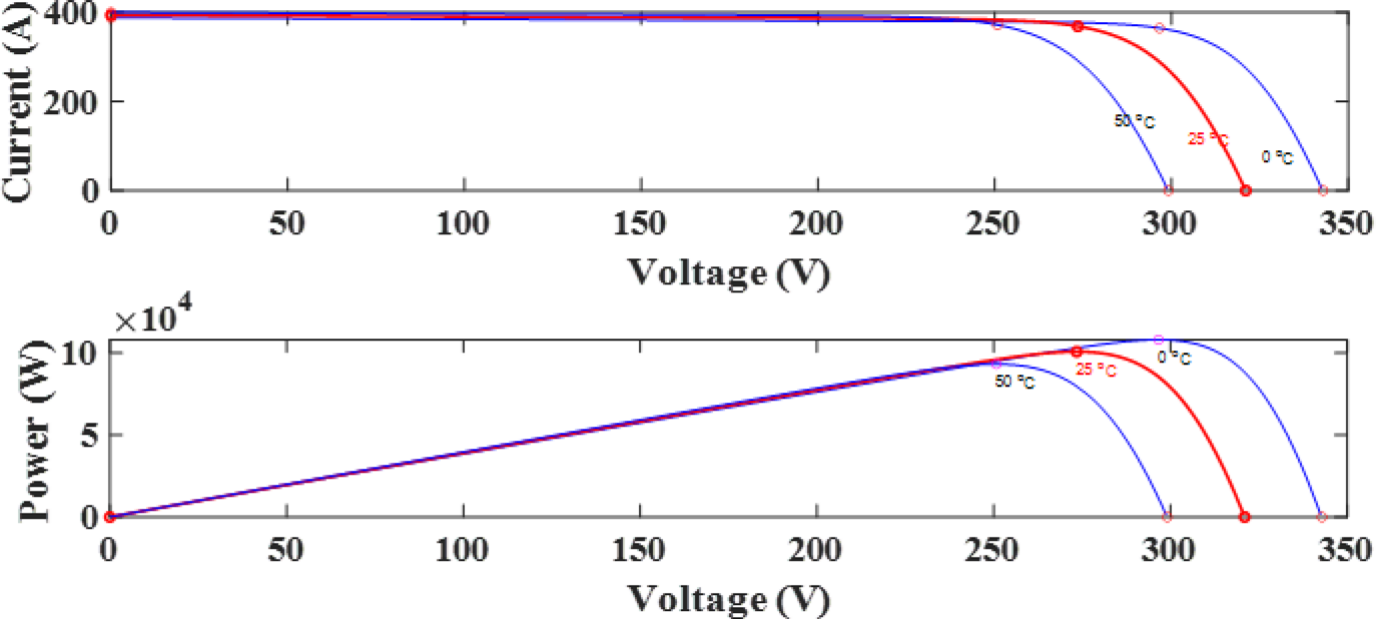
The I-V and P-V curves of PV output characteristics at various temperatures.

Figure 2 illustrates the equivalent circuit of the PV solar cell, providing an overview of the system’s fundamental electrical functioning. The PV model is used to generate the current-voltage (I-V) and power-voltage (P-V) curves, which are shown in Fig. 3. These curves are essential for understanding how the PV system behaves in various scenarios. Using an integrated regulator in conjunction with the incremental conductance approach, the PV module’s global MPPT is accomplished. By precisely tracking the highest power production, this strategy maximizes the system’s efficiency. Figure 5 illustrates ramp changes in the performance of a photovoltaic (PV) system under different conditions:

.

**Fig. 4:**
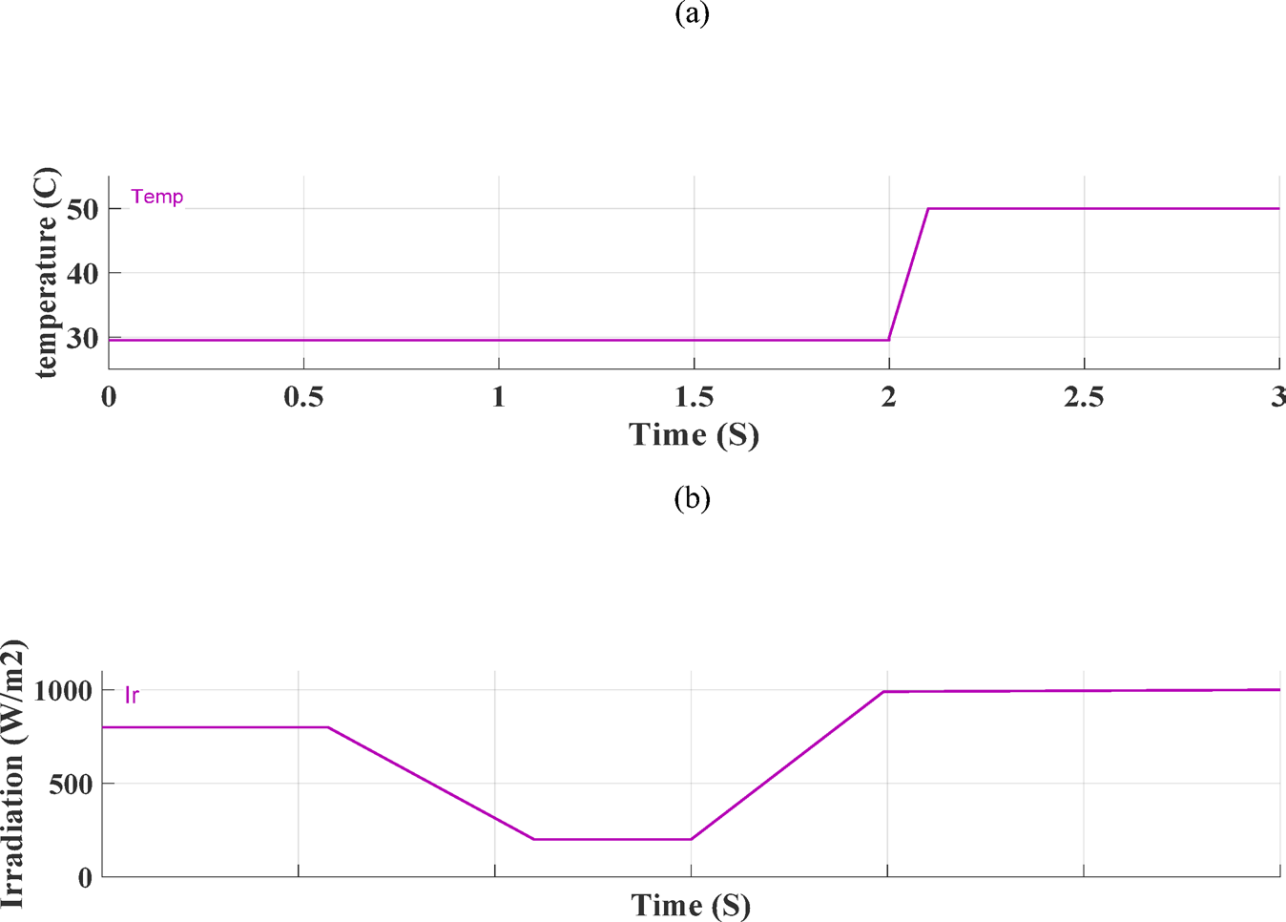
Ramp changes (**a**) various temperatures, and (**b**) various irradiations.

### DC-DC converter

**Fig. 5:**
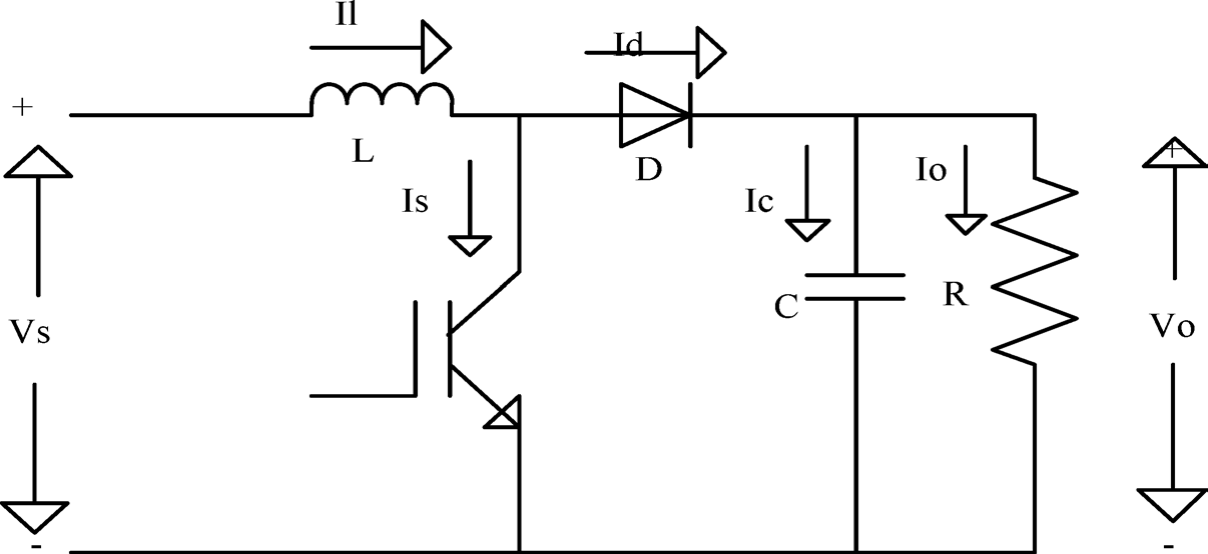
DC-DC Boost Converter.

To increase the DC voltage output from the PV panels in this system, a boost converter is employed as the DC-DC converter. The boost converter must first convert DC electricity to a higher DC voltage that the inverter can handle before it can be converted to AC power for grid integration. Figure 5 illustrates the fundamental layout of the boost converter, whose output voltage is determined by a specific equation. The switch, capacitor, diode, and inductor make up the boost converter. The inductor stores energy when the switch is closed. The stored energy is released when the switch is opened, resulting in an increase in the output voltage (VO). For the inverter to function well and provide grid-synchronized AC power, a greater voltage is required. By maintaining the proper voltage level for energy conversion, the boost converter is crucial for the efficient operation of the PV solar panel system^32^.

$$\:\text{V}\text{o}=\frac{\text{V}\text{s}}{1-\text{D}}$$

Where V_S_​ is the input voltage and D represents the duty cycle, which is the ratio of the on-time to the total switching cycle of the converter.

## Proposed controllers

**Fig. 6:**
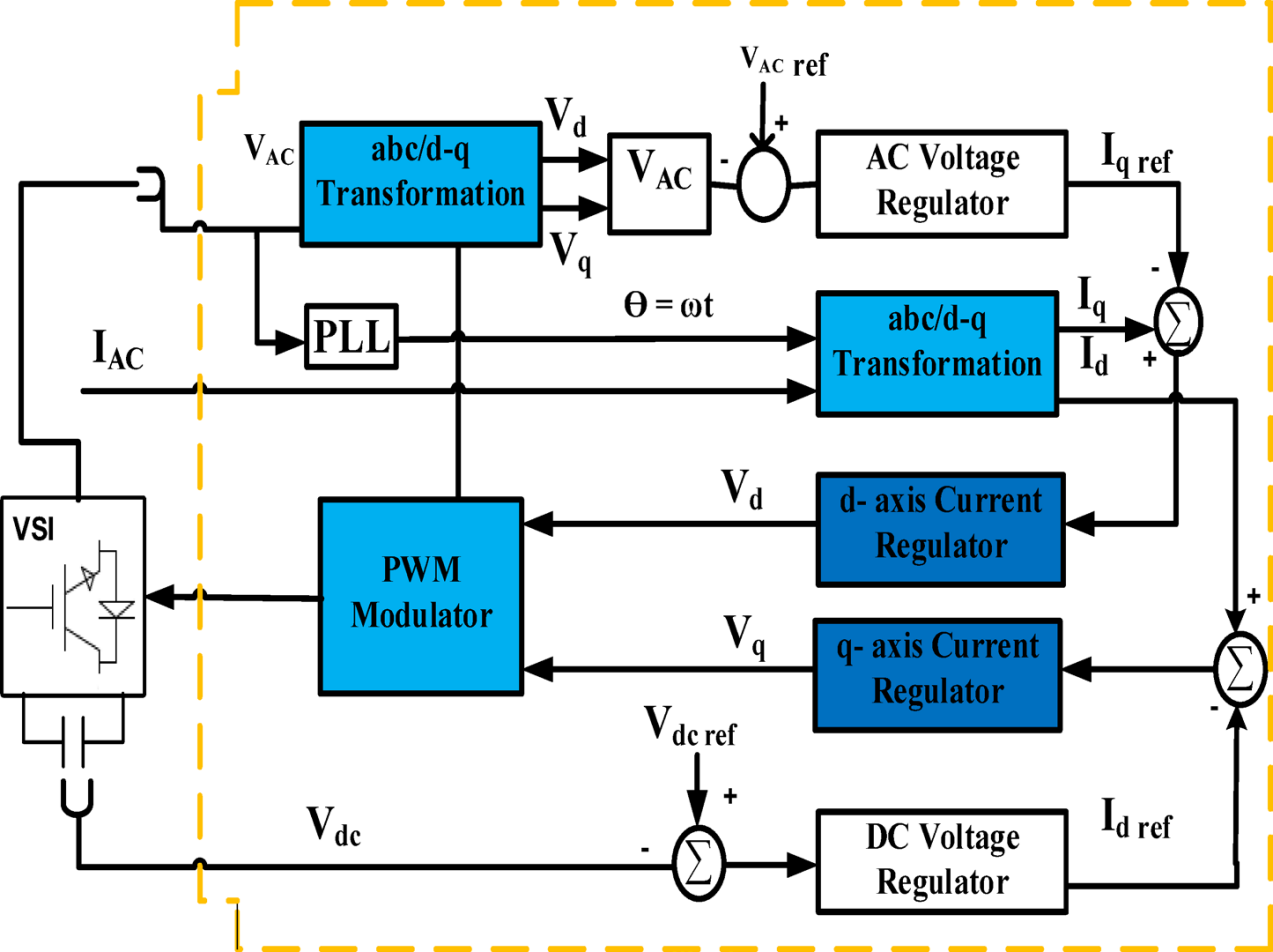
Proposed controllers with PV system.

**Fig. 7:**
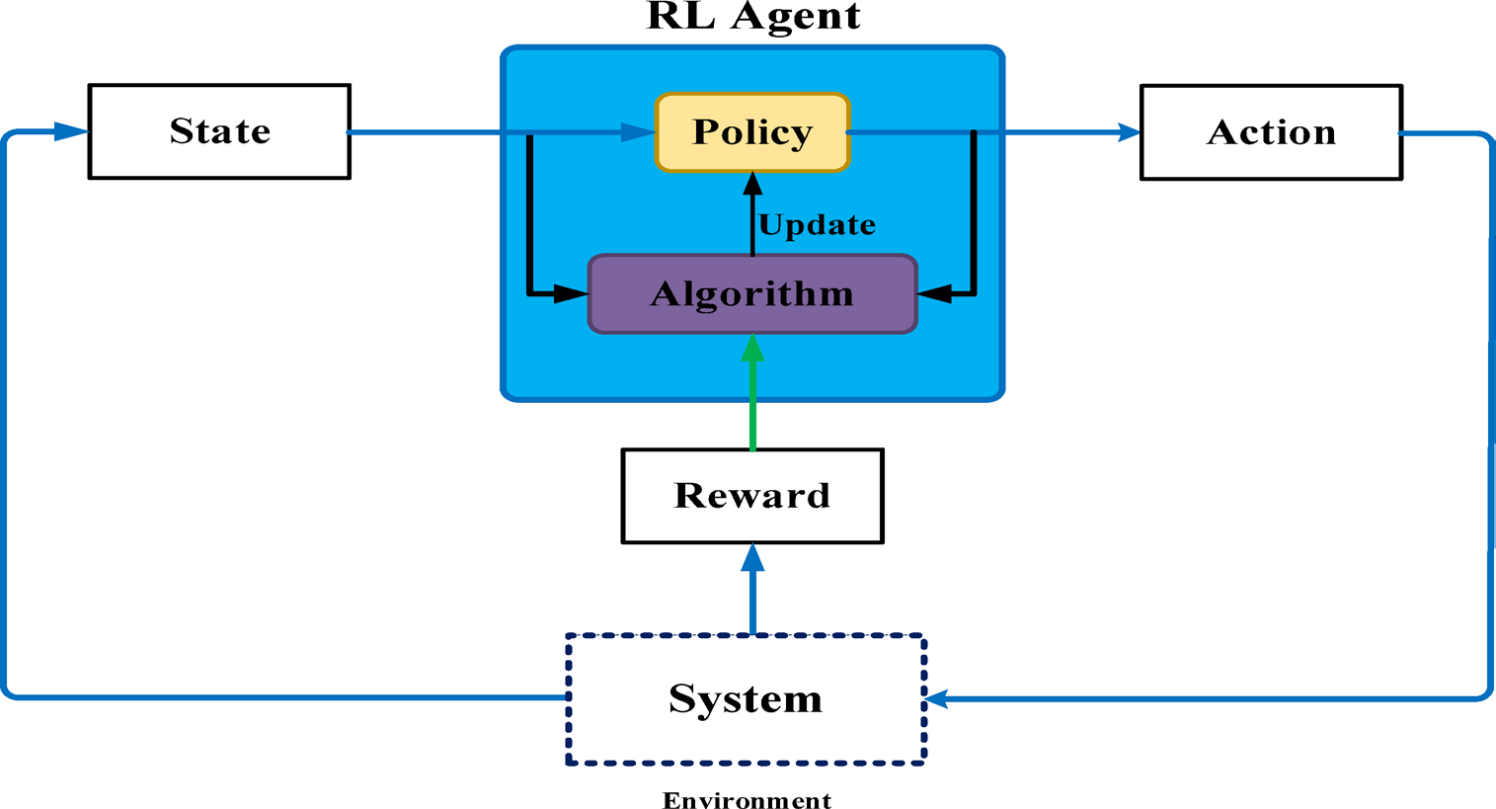
The controller module in the system’s inverter.

There are two methods of control in the inverter used in this work: two types of AI techniques. The RL type, particularly the second type, is ANN, which has gained traction in renewable energy applications for controlling utilization due to its ability to learn patterns, optimize processes, and make data-driven decisions. This work, which was studied, has applied AI algorithms such as NNs and evolutionary algorithms to optimize energy systems, including PV systems^31,32^. These approaches have shown promise in predicting energy output, detecting faults, and diagnosing systems, paving the way for more intelligent and autonomous PV systems. Figure 6 shows the PLL structure in the ANN controller. Figure 7 shows the controller block in the system’s inverter, which illustrates the training of the system using the RL method.

### Artificial neural network (ANN)

An element of both intelligent control and advanced control is ANN. The inverter in the RES is controlled by an ANN, a type of controller designed to tackle complex problems^33^. ANN control used in this study comprises a three-layer structure:

Input Layer: Consists of one input node receiving the system’s features.

Hidden Layer: This layer comprises 11 neurons with sigmoid activation functions, introducing non-linearity and enabling the network to model complex patterns in the data.

Output Layer: Contains one output node responsible for providing the control signal.

The ANN is trained using the Levenberg-Marquardt optimization algorithm to minimize the Mean Squared Error (MSE). The training results indicate a fast convergence, achieving an MSE of 0.00422 after nine epochs.

The sigmoid activation function, used in the hidden and output layers, is mathematically defined as:

9 $$\:a=\frac{G1}{1+{e}^{-n}}$$

**Fig. 8:**
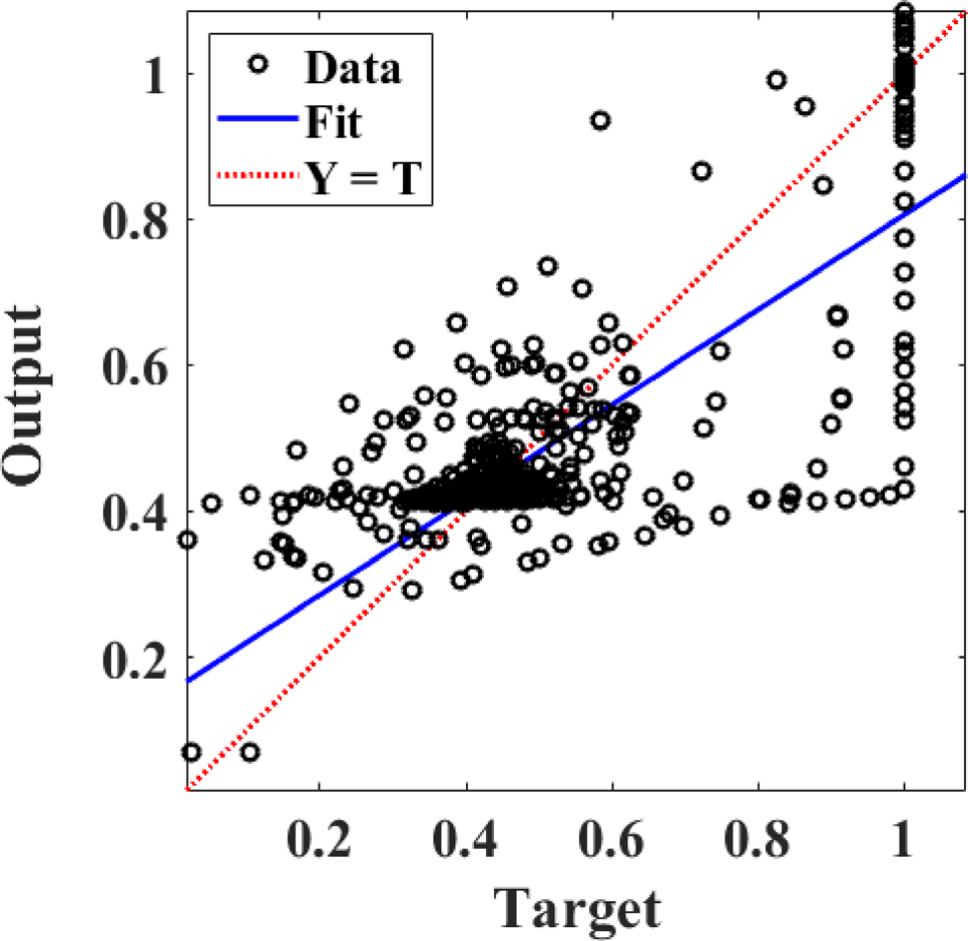
Neural network training.

**Fig. 9:**
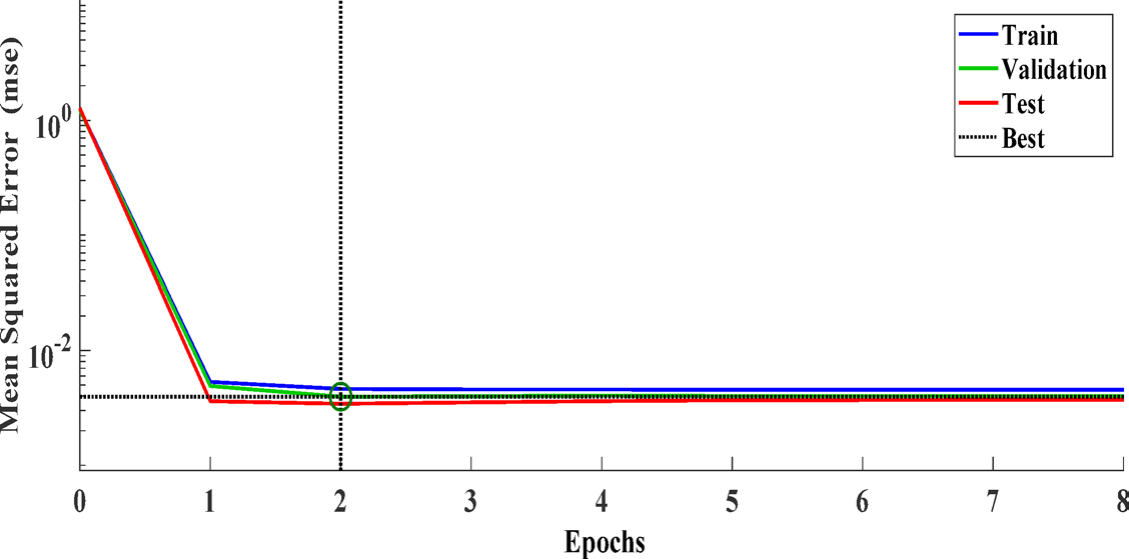
The best validation of ANN.

Training ANN value equal Training: *R* = 0.79933. From Fig. 8, all the data from the best training collected at the point between the two lines equal the best training at hidden layer 11. Figure 9 illustrates that the optimal validation performance is achieved at 0.003956, corresponding to epoch 2. From this figure, the training, validation, and test lines converge at the best point (2 on the x-axis) with a mean squared error of 0.0001.

**Fig. 10:**
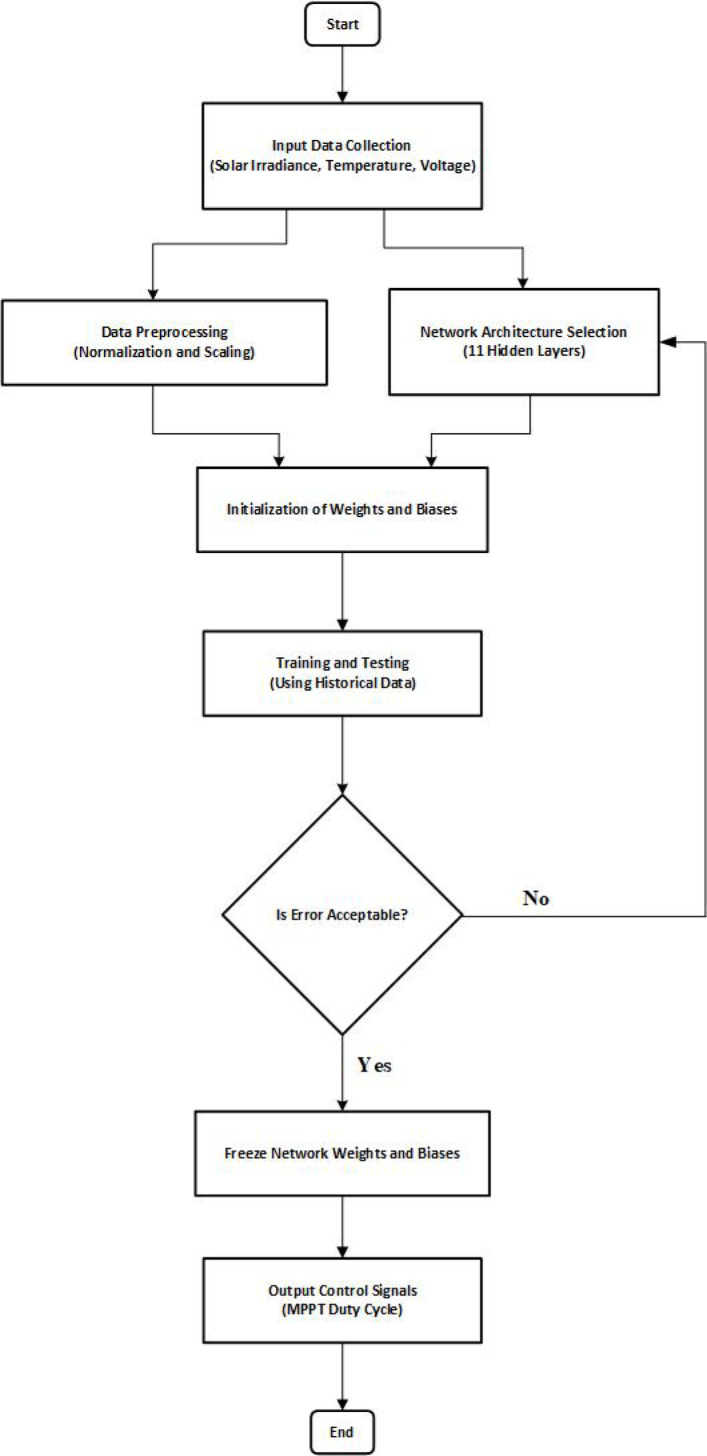
ANN-Based MPPT Controller Development Flowchart.

The design and implementation of an ANN-based controller for MPPT in a solar energy system are shown in this flowchart, as shown in Fig. 10. First, the input data, such as voltage, temperature, and sun irradiance, are gathered. Then the data is preprocessed (normalization and scaling). Eleven hidden layers comprise the chosen ANN design, and weights and biases are assigned. Historical data is used to train and test the network. The network weights and biases are completed, and control signals for the MPPT duty cycle are generated if the error is deemed acceptable. Otherwise, iterations are made until an acceptable error is reached.

### Reinforcement learning (RL)

RL is an exciting area of AI that enables agents to learn independently by interacting with specific environments. Together, RL and DL have demonstrated remarkable outcomes in several domains, including robotics, gaming, finance, business management, and natural language analysis. However, a significant challenge to RL is the impracticality of using look-up tables to manage massive state-and-action spaces in real-world scenarios. To get around this issue, NNs may be trained to connect states or state-action pairs with Q-values by estimating value or policy functions^34^.

**Fig. 11:**
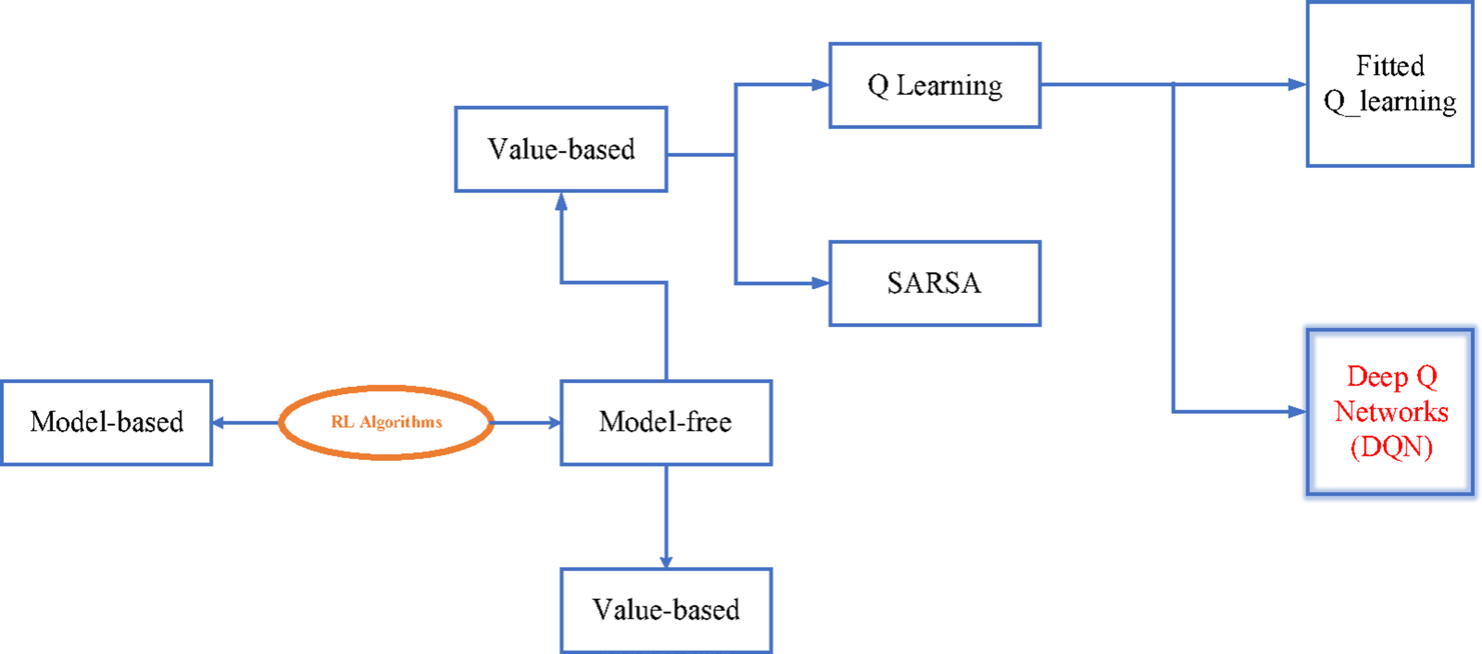
Introduction of RL (RL) algorithm.

Model-based RL entails either comprehending or learning about the model, as seen in Fig. 11. The effectiveness of this approach in learning from a small number of examples is a significant benefit. But when the model is hard to train, it becomes computationally demanding. In comparison, model-free RL is more realistic as it doesn’t need a precise picture of the world to work and requires less processing power. There exist two types of model-free RL approaches: value-based and policy-based. The goal of value-based RL is to enhance the value function iteratively until it satisfies the convergence requirement^35^.

By developing a plan or policy, the agent gains knowledge on how to achieve the highest possible overall reward after completing an episode. As a result, we reward the agent positively when it makes the right decision and negatively when it performs poorly^36–38^. To utilize RL and DRL in an MPPT control, it is necessary to specify the reward and calculate the preset state and action spaces. Power, duty cycle, voltage, and current together comprise observation.

**Fig. 12:**
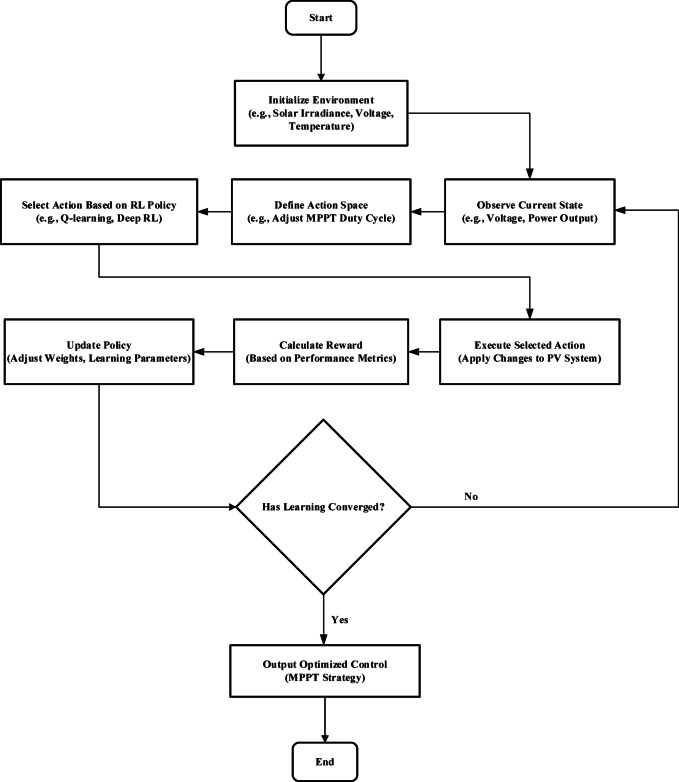
RL-Based MPPT Strategy Optimization Flowchart.

Figure 12 shows the flowchart that describes how to optimize the MPPT in a PV system using an RL-based approach. First, the environment (such as temperature, voltage, and solar irradiance) is initialized, and the present condition (such as voltage and power output) is observed. The MPPT duty cycle is then changed to define the action space. An RL policy (such as Q-learning or Deep RL) is used to determine the best course of action, which is then implemented in the PV system. Based on how well the system performs, a reward is determined, and the policy is modified (by changing learning parameters and weights). The optimal MPPT control method is produced at the end of this cycle, which continues until the learning converges.

A. Methodology of the DQN Control:

When exact models are unavailable, RL is very helpful in solving optimal control issues. However, because feature representation is a barrier, RL struggles with high-dimensional or continuous action spaces. Deep Q Networks (DQN) combine Q-learning and deep-learning methods to address this problem. With DQN, the conventional Q-table is replaced with a deep neural network (Q-network) that approximates the Q-function to forecast future rewards by mapping environmental conditions to agent behaviors. Two essential elements improve the learning process. To smooth data distribution and lessen correlation, an agent’s experiences are first recorded in a replay buffer. Secondly, to lower the mean squared error between the desired Q values and the predictions, transitions are randomized in mini-batches from this buffer. The Q-network is updated in real-time, whereas the target network is static for a predetermined number of steps until its weights are modified by copying information from the Q-network. Training process stability is aided by this set duration for the target network.

The mathematical framework includes:

- Q-Learning Update Rule:

$$\:Q\left(s,a\right)=R+{\gamma\:max}_{a}\:Q({s}^{{\prime\:}},a)$$

- Neural Network Approximation: The Q-function is approximated by a neural network that maps states and actions to predicted rewards. The network is trained to minimize the loss:

$$\:L=\text{{\rm\:E}}\left[\right(Q\left(s,a\right))-(R+{\gamma\:max}_{a}\:Q({s}^{{\prime\:}},a)\left){)}^{2}\right]$$

**Fig. 13:**
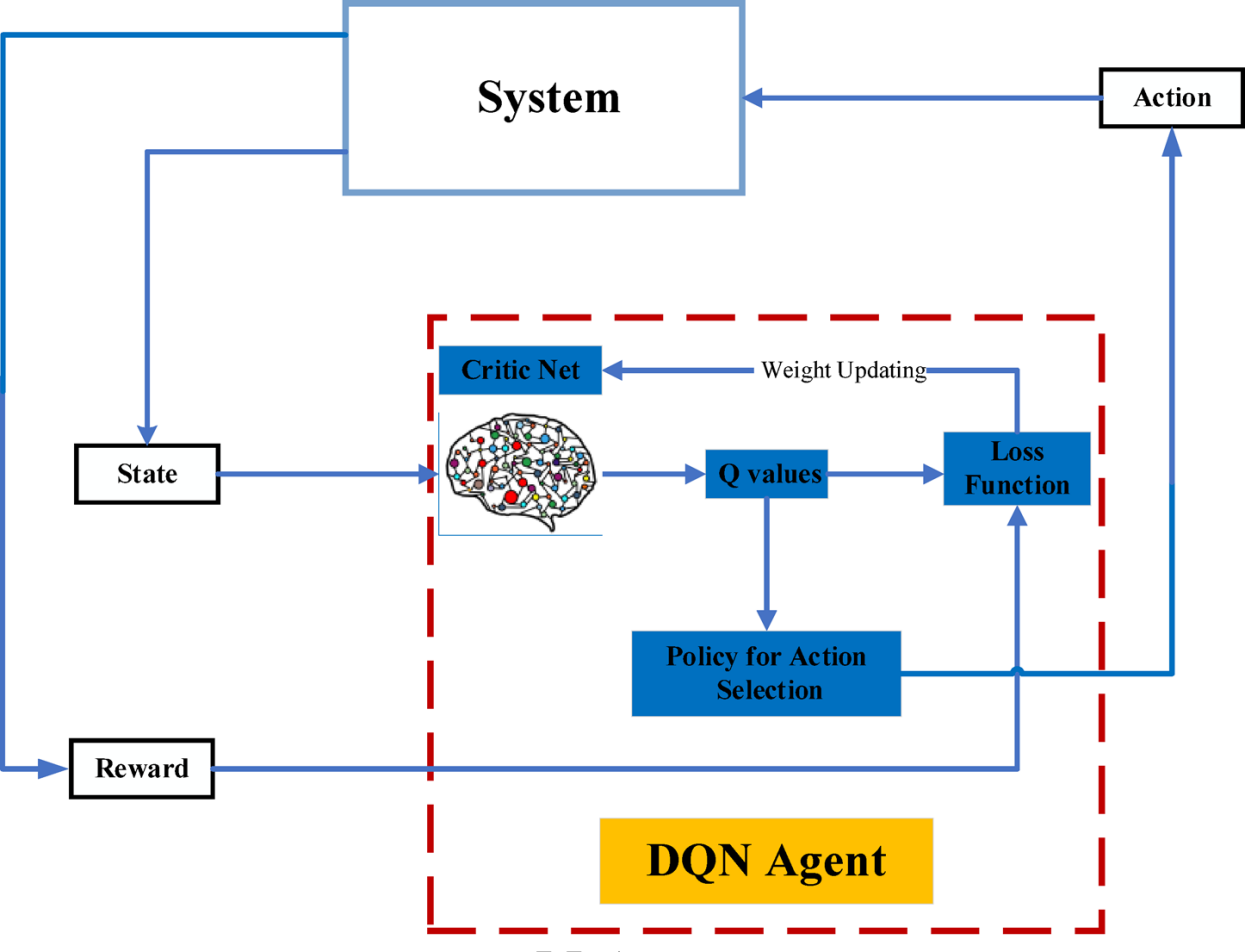
A deep Q network (DQN) algorithm diagram.

Figure 13 provides a schematic that depicts the DQN technique.

**Fig. 14:**
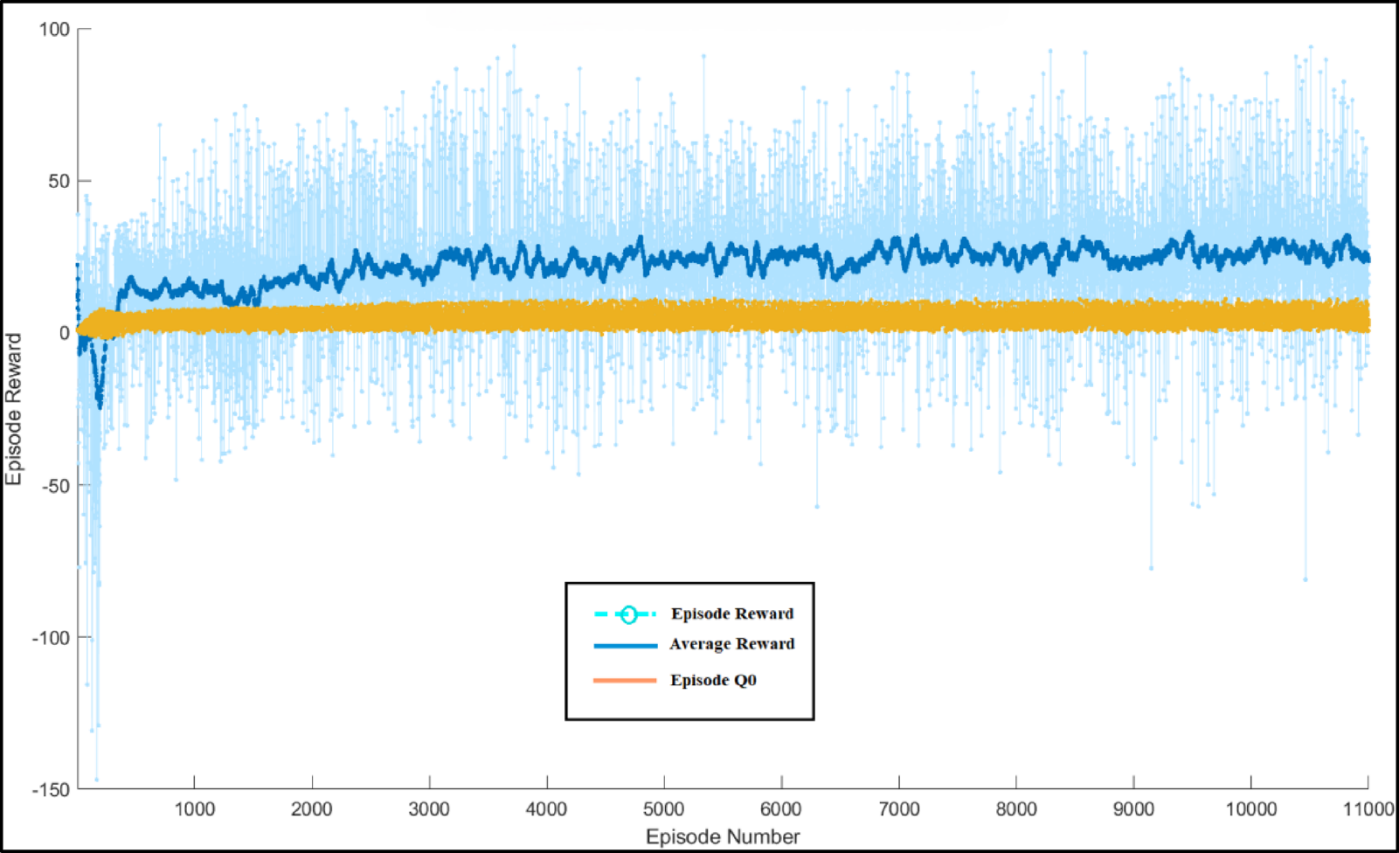
Training process of the DQN method.

B. Training Results:

Figure 14 displays the training outcomes of the DQN approach. During the training phase, the DQN agent retains all interaction data, such as reward, state, action, and subsequent state, in memory. At each step, a tiny batch of this memory is randomly sampled to train and update the NN weights. The graph’s blue line represents the cumulative reward for each episode (Episode Reward), while the indigo line chart shows the average reward value during the training period. The orange line is for Episode Q0. After training, the parameters of the DQN agent are kept for use in online control applications, and their ability to interact with the environment is employed to verify them. Figure 14 illustrates that a range of input conditions are available. During the training phase, the DQN agent retains all interaction data, such as reward, state, action, and subsequent state, in memory. At each step, a tiny batch of this memory is randomly sampled to train and update the NN weights.

## Simulation results and discussions

### Comparison results under ramp changes in solar radiation

The specifications for the full microgrid are presented in Table 2, along with the features of the PV solar panel system, the component values of the boost converter, and essential output metrics, including grid voltage regulation, current, and power. The controllers can successfully regulate and optimize PV system performance in the face of shifting environmental elements and system demands by assessing them under these diverse settings.

**Table 2:** System parameters.

Parameter	Value
*PV Parameters*	
Maximum Power of PV Module	305.226 W
Open Circuit Voltage	64.2 V
short circuit current	5.96 A
Maximum voltage	54.7 V
Maximum current	5.58 A
PV array parallel strings	66
Series-connected modules per string	5
The inductance of the boost converter	5e-3 H
Boost converter resistance	0.005 Ὡ
Boost converter capacitance	100e-6 F
Total P_PV_ Array	100 KW
*Grid Parameters*	
Step up transformer	260 V/25 kV
Voltage of the grid	20,000 V
Frequency of grid	60 Hz
Output current grid at ANN	4 Amp
Output current at RL	6 Amp
Active power in ANN	82 KW
Active power in RL	100 KW

**Fig. 15:**
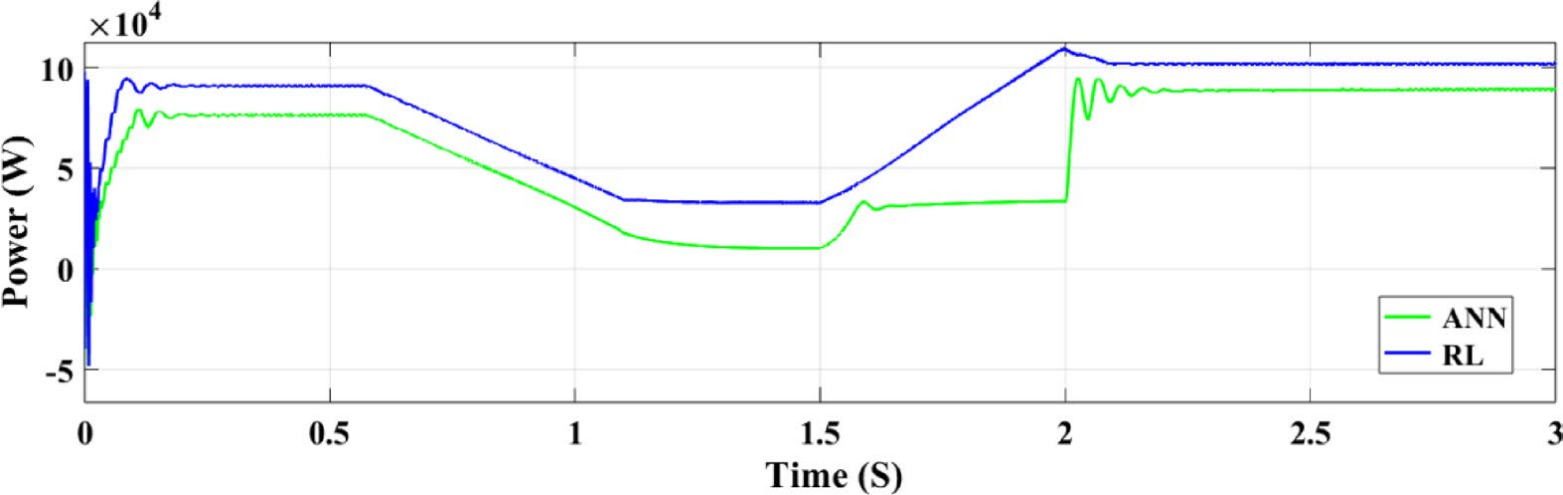
PV solar panel output power.

**Fig. 16:**
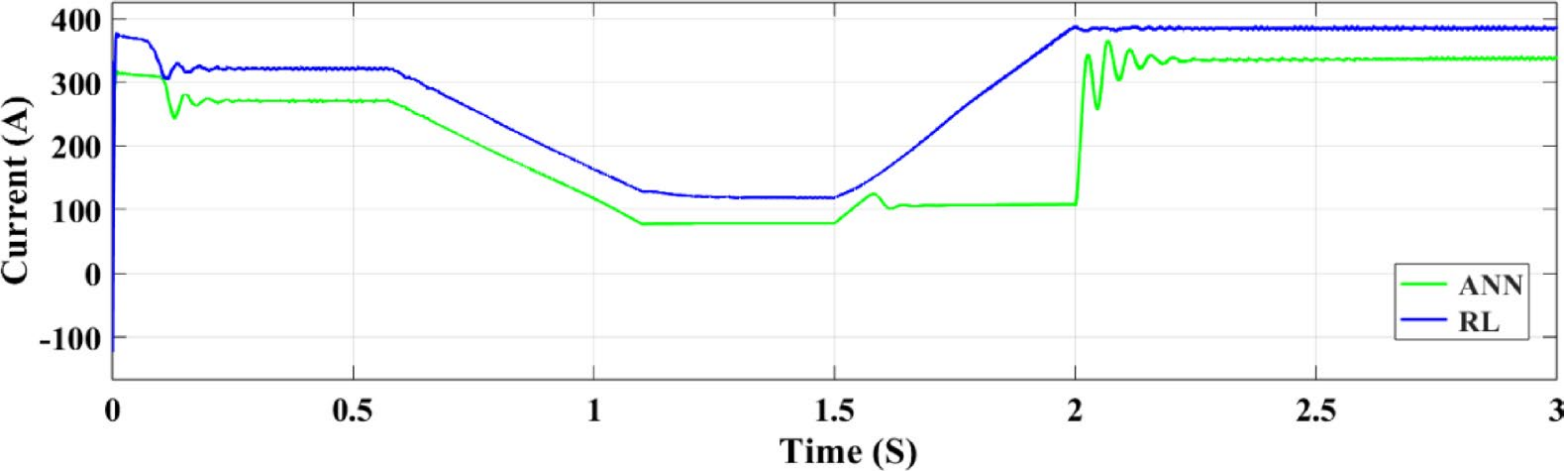
PV solar panel current at two controllers.

**Fig. 17:**
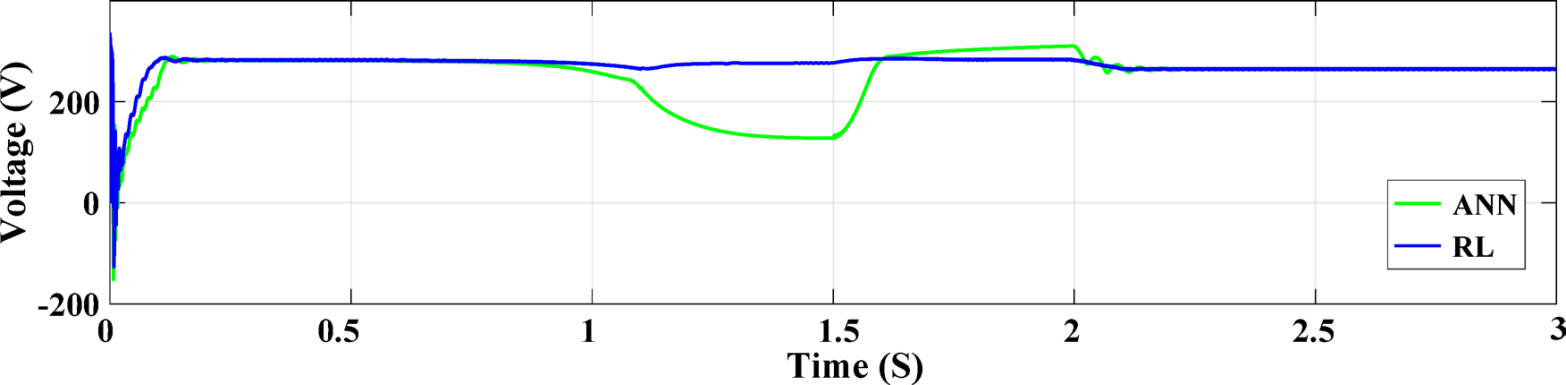
PV solar panel voltage at two controllers.

In the system described in Fig. 1, the output PV solar panel power for both ANN and RL controllers is shown in Fig. 15. With its effective operation and minimal ripples, the RL controller consistently generates satisfactory results in terms of PV power generation. In contrast, the ANN controller performs poorly between 1.5 and 2.25 s, with a lower output and greater power ripples, despite overall performance being satisfactory. In Fig. 16, the PV current for both controllers are shown. The ANN controller exhibits noticeable ripples and an uneven current supply within the 1.5–2.25-second interval, demonstrating poor performance once again. The RL controller, however, provides reliable and respectable current performance. The PV voltage output for both controllers is displayed in its effective operation and minimal ripples, the RL controller consistently generates satisfactory results in terms of PV power generation. In contrast, the ANN controller performs poorly between 1.5 and 2.25 s, with lower output and greater power ripples, even if it works well overall. In Fig. 17, the PV voltage for both controllers are shown. The ANN controller exhibits noticeable ripples and an uneven current supply within the 1.5–2.25-second interval, demonstrating poor performance once again.

**Fig. 18:**
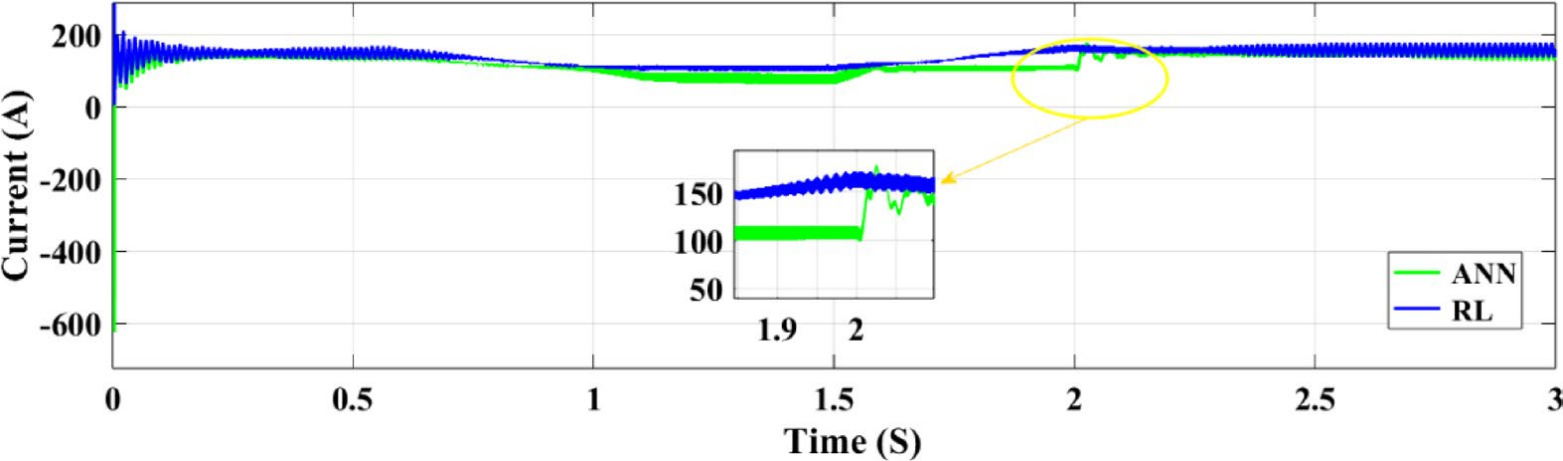
The DC-DC boost converter for both controllers’ current output.

**Fig. 19:**
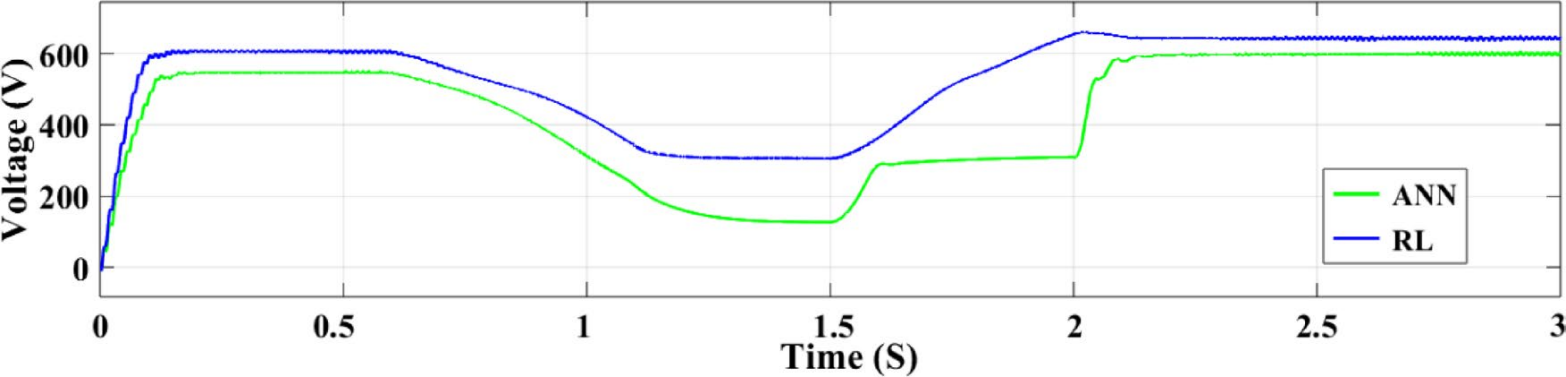
The DC-DC boost converter for both controllers’ voltage output.

**Fig. 20:**
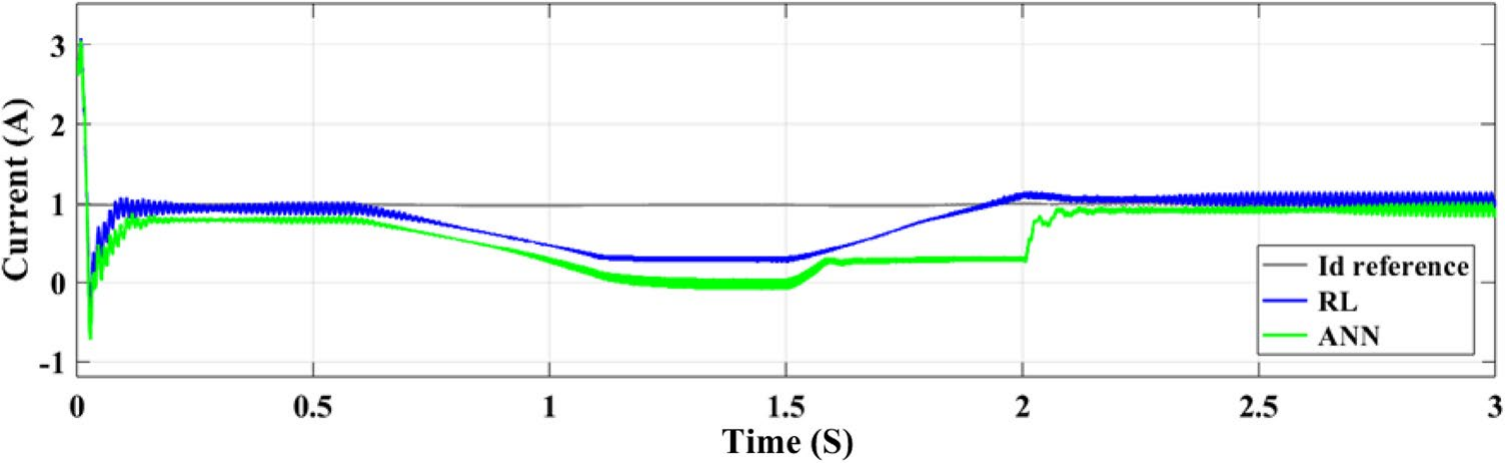
Injected Current control at two Controllers.

The DC-DC output current of the converter is illustrated in Fig. 18. The ANN controller performs worse than the RL controller when the time interval between 1.8 and 2.2 s is zoomed in on. However, the ANN controller exhibits undershooting, reduced current, and noticeable ripples. In contrast, the RL technique maintains a more constant current output. The voltage output of the boost converter of the DC-DC type with both controllers is illustrated in Fig. 19. All things considered, the ANN controller performs better than the RL approach, even if it produces outcomes that are generally acceptable. The ANN controller exhibits fewer ripples throughout the 1.5–2 s period, while the RL method routinely produces better outcomes. The injected current control for both controllers is displayed in Fig. 20. When compared to the ANN Controller, the RL controller exhibits superior alignment with the intended performance,

typically following the reference current line closely.

**Fig. 21:**
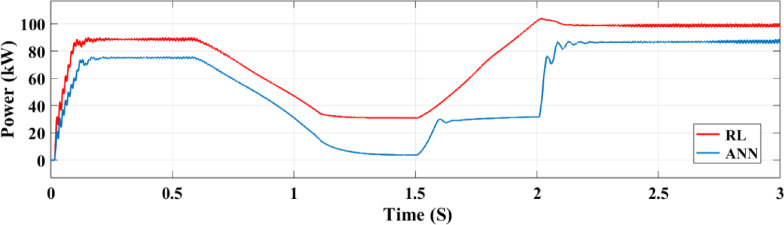
Analysis of the active grid power performance with two controllers.

**Fig. 22:**
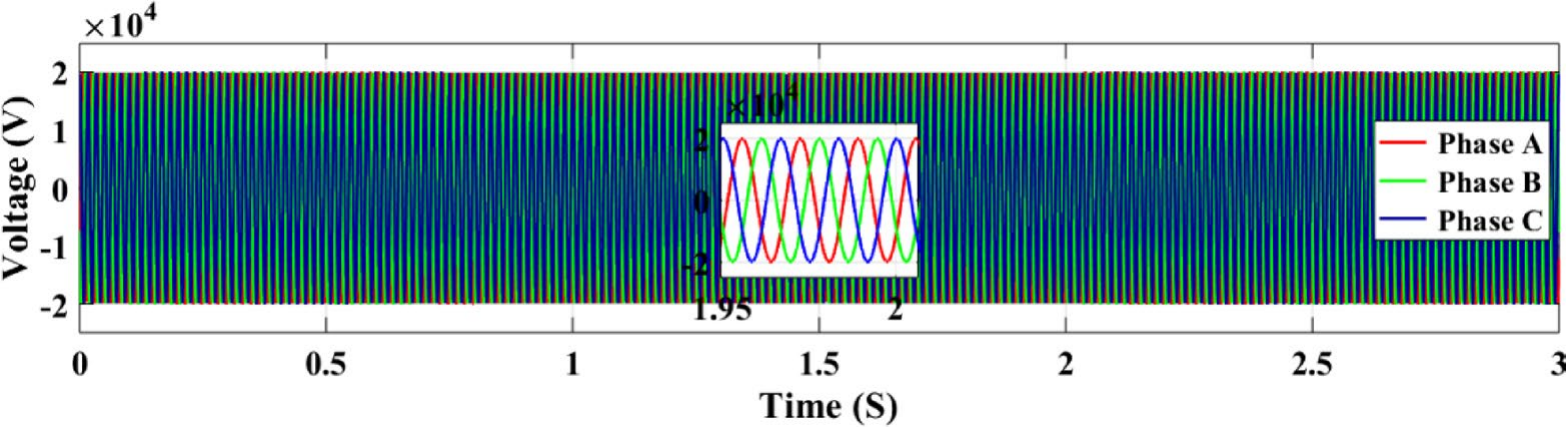
Output grid voltage profile.

The active power output of the PV solar panel system to the grid is illustrated in Fig. 21. In comparison to the ANN controller the RL approach yields a greater active power value of 100 kW while the ANN controller only manages to produce 82 kW. The temperature and irradiation variations occur between 0.5 and 2 s, during which the RL controller operates more effectively and sustains a more consistent power output. On the other hand, the ANN controller performs worse from 1.5 to 2 s and exhibits a drop in power output between 1 and 1.5 s. The output voltage of the inverter to the three-phase power grid is illustrated in Fig. 22. When the grid maintains regulation, both controllers provide an output voltage of 20 kV.

**Fig. 23:**
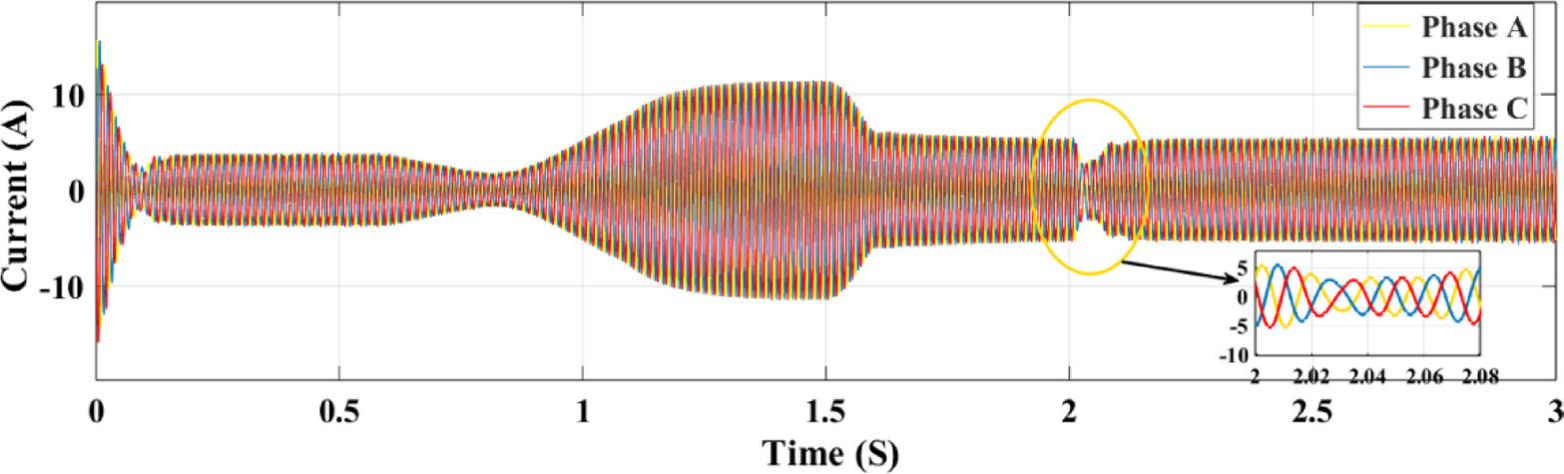
Output grid current using ANN.

**Fig. 24:**
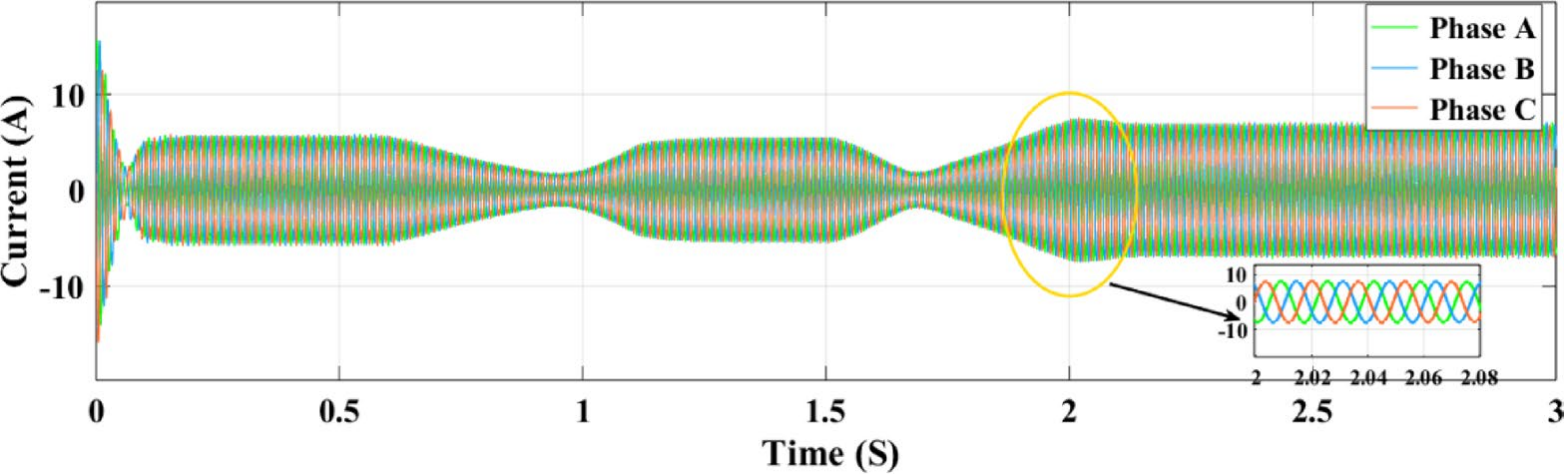
Output grid current using RL.

Figures 23 and 24 show the grid-injected current for the ANN and RL controllers, respectively. Compared to the ANN, the RL controller performs better and exhibits fewer overshoots. While the ANN controller only produces four amps of output current, the RL controller produces six amps. More current is sent to the grid by the RL controller as a result. When focusing on the period between 2 and 2.08 s, the RL controller performs noticeably better than the ANN controller, exhibiting greater grid current management capabilities. In comparison to the ANN controller, the RL controller provides more consistent and adequate power and current values for the microgrid, which makes it a better option for inverter control in a PV system.

Table 3 presents a detailed comparison between the ANN and RL control methods in a grid-connected PV system subjected to ramp changes in solar irradiance. The table includes key metrics such as grid power output (P_Grid_), PV power output (P_PV_), PV voltage (V_PV_), the corresponding irradiance level, and the control technique used.

The results clearly show that, in every operational scenario, the RL-based controller performs noticeably better than the ANN-based controller. The ANN controller achieves only 33.15 kW and 31.75 kW at high irradiance (1000 W/m²), whereas the RL controller obtains 109.66 kW of PV power and 102.25 kW of grid power, respectively, representing gains of more than 230% in PV power and 220% in grid power. Furthermore, at the same irradiance, RL delivers a PV voltage of 282.12 V as opposed to 239.22 V with ANN, maintaining superior voltage stability. Even at low irradiance levels (200 W/m^2^), RL exhibits more flexibility, generating outputs that are more stable and powerful. The learning capacity of RL, which permits dynamic policy updates and improved adaptability to environmental unpredictability, is responsible for this performance disparity. The ANN controller only reaches 33.15 kW and 31.75 kW at high irradiance (1000 W/m^2^), but the RL controller obtains 109.66 kW of PV power and 102.25 kW of grid power, respectively, which represents gains of more than 230% in PV power and 220% in grid power. Furthermore, at the same irradiance, RL delivers a PV voltage of 282.12 V as opposed to 239.22 V with ANN, maintaining superior voltage stability. Even at low irradiance levels (200 W/m^2^), RL exhibits more flexibility, generating outputs that are more stable and powerful. The learning capacity of RL, which permits dynamic policy updates and improved adaptability to environmental unpredictability, is responsible for this performance disparity.

**Table 3:** Comparative of the main results for ANN and RL under ramp changes in solar radiation.

Controller	Irradiance (W/m^2^)	Time (s)	V_PV_ (V)	*P*_PV_ (kW)	*P*_Grid_ (kW)
ANN	800	0.13	282.19	70.25	72.35
	200	1.5	127.55	9.96	3.73
	1000	2	309.93	33.15	31.75
RL	800	0.13	288.97	91.11	84.67
	200	1.5	276.18	32.83	31.09
	1000	2	282.12	109.66	102.25

**Fig. 25:**
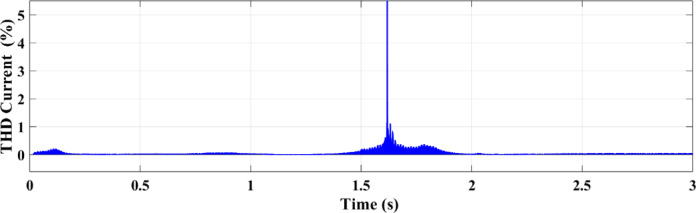
THD of the current under ramp condition with ANN.

**Fig. 26:**
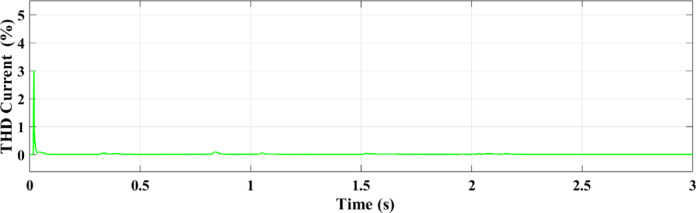
THD of the current under condition ramp with RL.

Figures 25 and 26 illustrate the THD of the current under a ramp input condition using ANN and RL, respectively. In Fig. 25, the ANN-based controller exhibits a noticeable spike in THD at approximately 1.55 s, where the distortion briefly exceeds 5%. This peak indicates a moment of instability or high-frequency distortion, suggesting that while the ANN controller is adaptive, it may introduce transient nonlinearities or struggle with smooth dynamic transitions under certain conditions. Conversely, Fig. 26 demonstrates the performance of the RL-based controller, which maintains a significantly more stable response. The THD remains consistently below 1% throughout the 3-second interval, reflecting superior harmonic control and reduced current distortion.

**Fig. 27:**
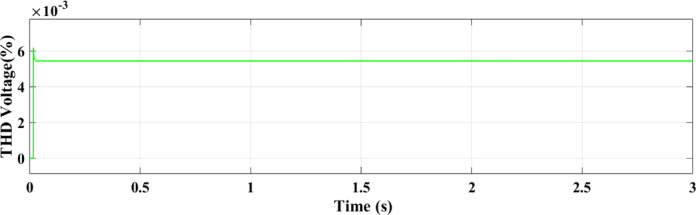
THD of the voltage with ANN.

**Fig. 28:**
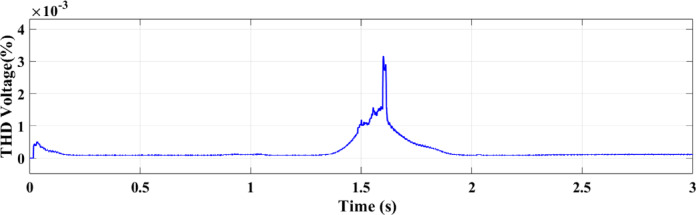
THD of the voltage with RL.

Figure 27 shows the THD of the voltage under ramp solar radiation using the ANN controller. Its capacity to adjust to shifting environmental circumstances is confirmed by the RL controller’s THD for voltage under ramp conditions, which shows better voltage regulation and fewer distortions as shown in Fig. 28.

### Comparison results with random variations in solar radiation

**Fig. 29:**
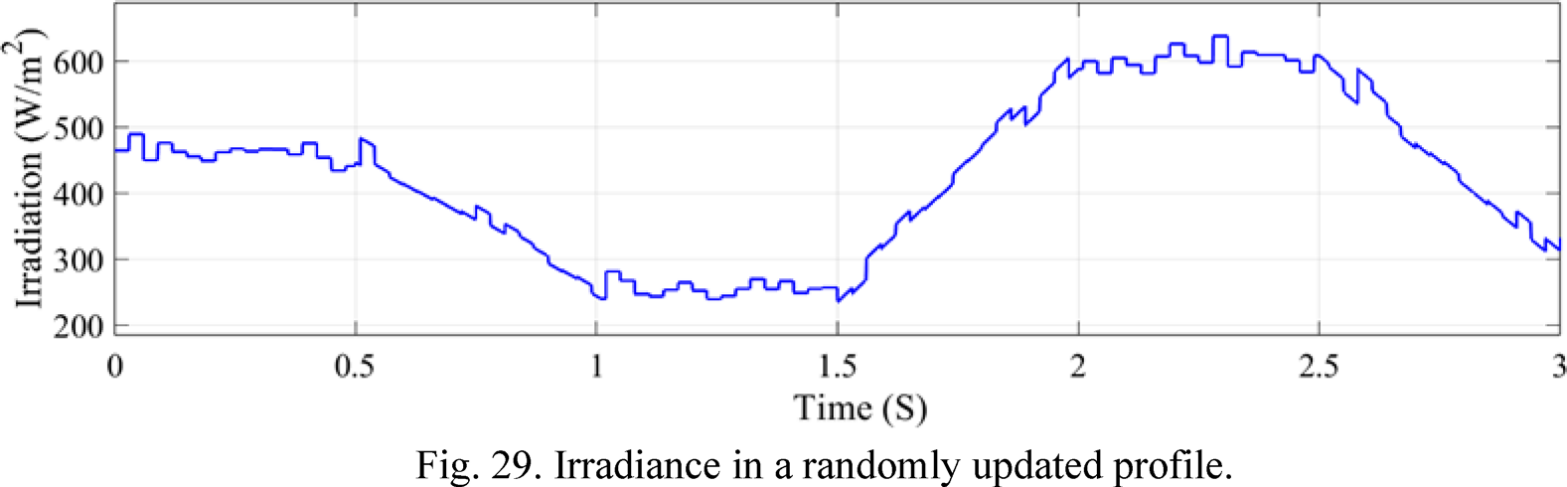
Irradiance in a randomly updated profile.

A model incorporating random fluctuations in solar radiation and a mean value of 500 W/m² is displayed in Fig. 29. In this model, the outside temperature is kept constant. Rainy weather and cloud cover are two examples of the variables that might contribute to these variations in solar energy. Maintaining the efficacy of the suggested control system and guaranteeing dependable operation in a range of environmental circumstances depends on effectively managing these erratic variations in radiation.

**Fig. 30:**
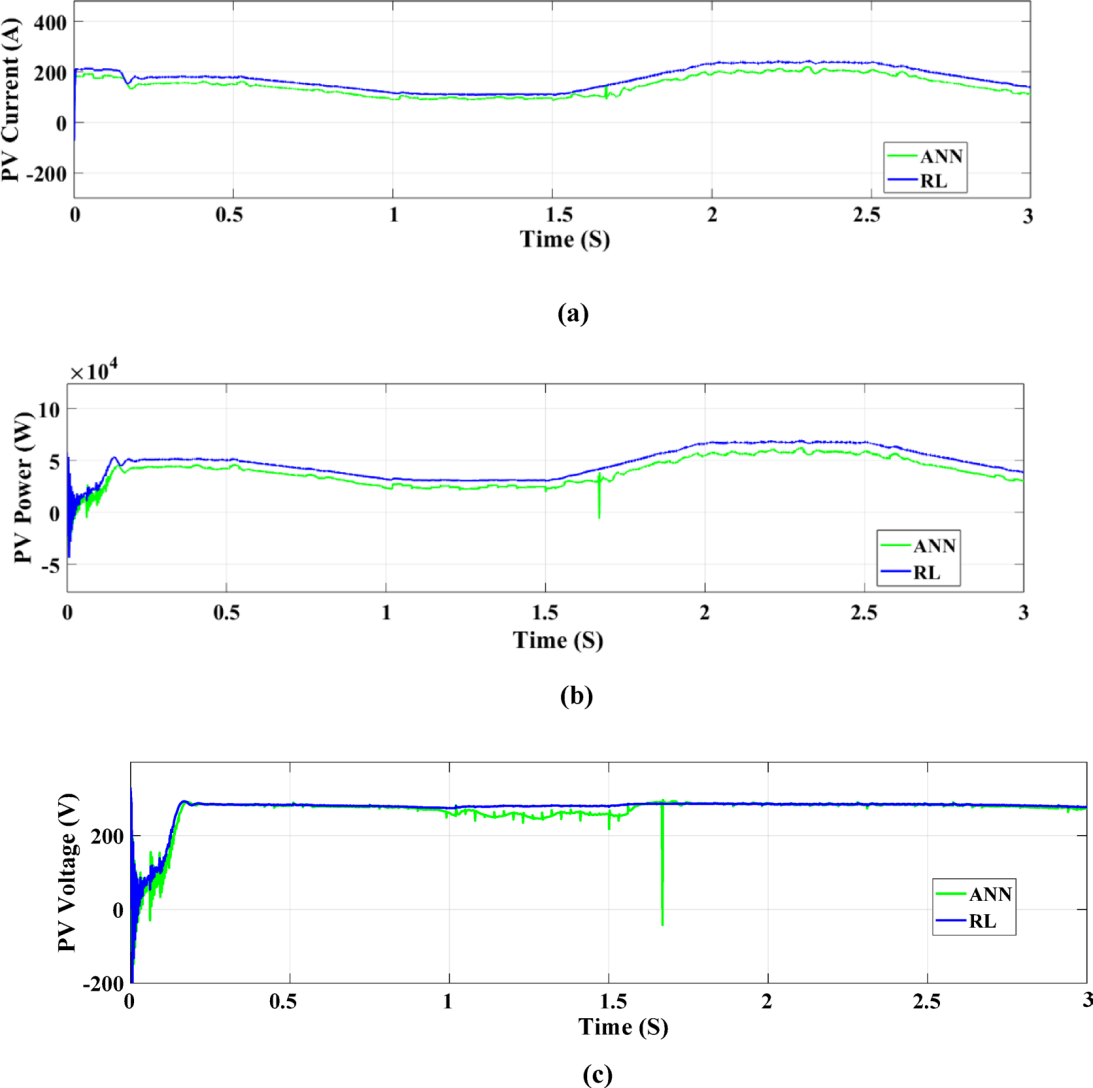
DC performance results of a PV solar panel using both control systems (RL and ANN). With Random Variations in Solar Radiation. (**a**) PV solar panel output current, (**b**) PV solar panel output power, and (**c**) PV solar panel output voltage.

In Fig. 30, two control systems—RL and ANN—are used to compare the maximum production of PV electricity under fast random changes in solar radiation. In terms of controlling current output during these variations, the RL control system performs noticeably better than the ANN method. Figure 30 A illustrates that the RL control system displays a greater current output than ANN between 0 and 0.5 s at a solar radiation intensity of 460 W/m². The ANN system delivers 155 amperes of current, while the RL system maintains 180 amperes while radiation drops below 250 W/m² over 0.5 to 1 s. The current of the RL system climbs to 245 amps while that of the ANN system stays at 210 amps when radiation levels reach 600 W/m² for 1.5 to 2 s.

Significant current instability is seen in ANN during this period. ANN obtains a maximum current of 200 amperes during the stabilization phase, whereas RL achieves a maximum current of 240 amperes, during the 2 to 2.5 s period. The RL current lowers to 160 amperes and the ANN dips to 118 amperes as radiation declines from 2.5 to 3 s to 310 W/m². Comparing the ANN system with the RL control system, Figure 30B shows that the RL control system offers more stability and maximum power output under random radiation variations. This is most noticeable when radiation maximizes from 250 W/m² to 600 W/m², which occurs between 1 and 2 s. When it comes to controlling PV power output amid these abrupt fluctuations in solar radiation, the RL system often beats the ANN system.

The voltage variations in PV cells under ANN and RL system management are shown in Fig. 30C. In contrast to the ANN system, the RL control system attains a more steady voltage despite some early oscillations. Both systems settle at 285 volts after 0.5 s, but the RL system keeps the voltage higher and more constant throughout that time. This suggests that, particularly in situations with rapidly fluctuating weather, the RL system consistently outperforms the ANN control system in raising the output voltage of the PV solar panel cells.

**Fig. 31:**
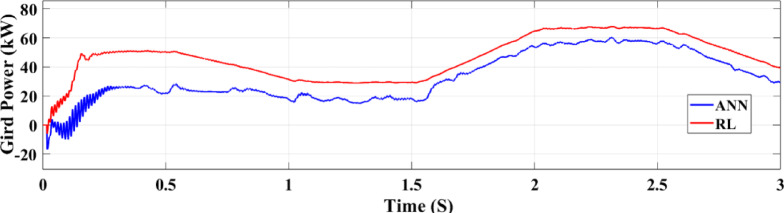
Grid power under random irradiance.

Figure.31. depicts the grid power output for both RL and ANN control systems under random solar radiation. The RL system not only produces higher power but also maintains greater stability, demonstrating superior efficiency in utilizing the PV power system compared to the ANN system.

**Fig. 32:**
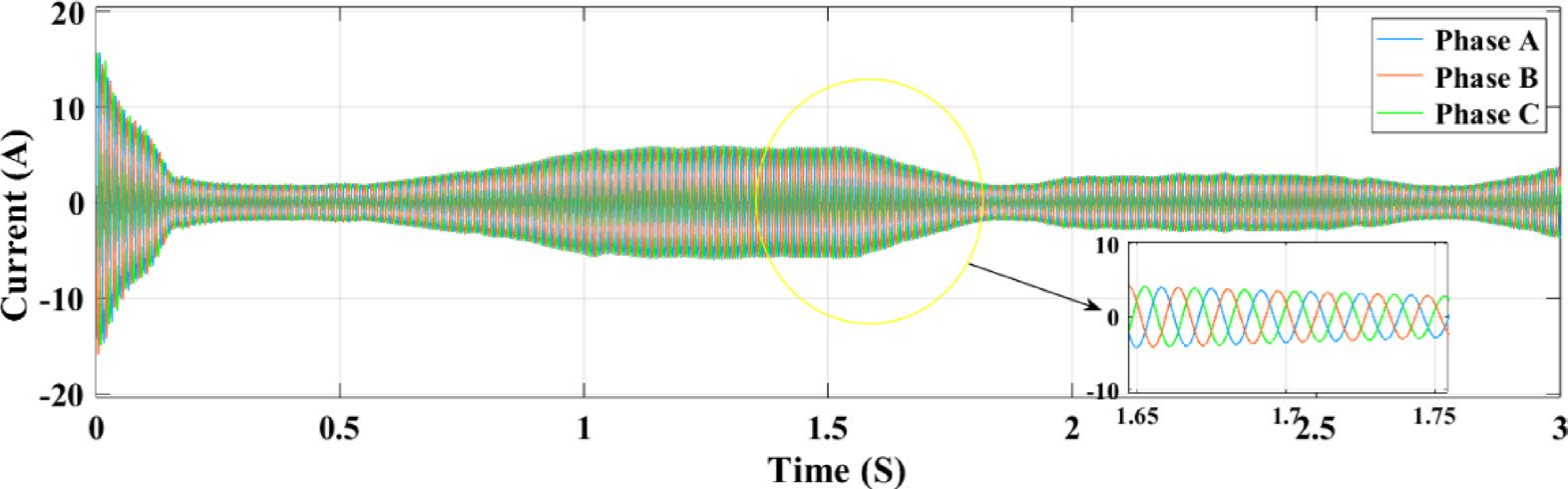
Grid current under random irradiance using RL.

**Fig. 33:**
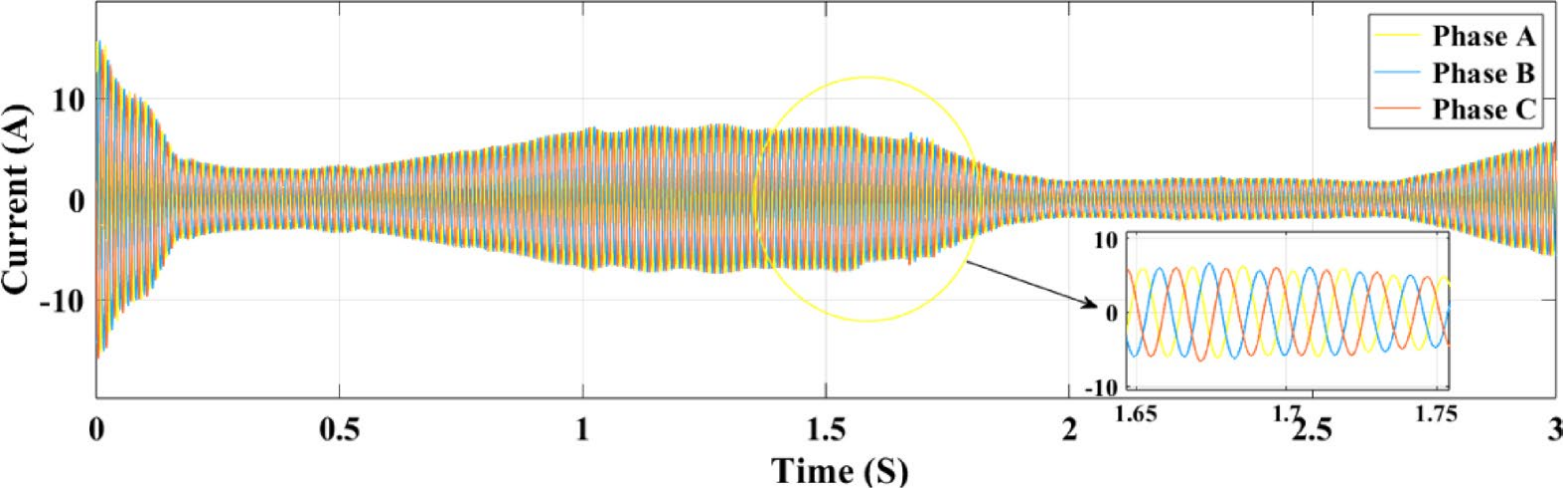
Grid current under random irradiance using ANN.

Figure 32 illustrates the association between the three-phase current and the random fluctuations in solar irradiance with the RL controller, tracking the top power level for grid feeding. The present variation is depicted as it moves through the ANN control system in Fig. 33 and as it moves through the RL system in Fig. 32. The relationship between the three-phase currents in the PV solar panel system and fluctuations in solar radiation is seen in Figs. 32 and 33. Time is plotted on the x-axis, while the y-axis illustrates the values at present. Phase B currents are represented by the blue line, phase C currents by the red line, and phase A currents by the yellow and green lines.

The conversion of variations in solar energy into variations in current is seen in Fig. 32. It records the highest power point that may be delivered to the grid by showing the link between the three-phase current and the random variations in solar irradiation. Figures 32 and 33 show the current fluctuation as it passes through the ANN control system and the RL system, respectively. It is shown that the RL system responds faster and more accurately than the ANN control system.

**Fig. 34:**
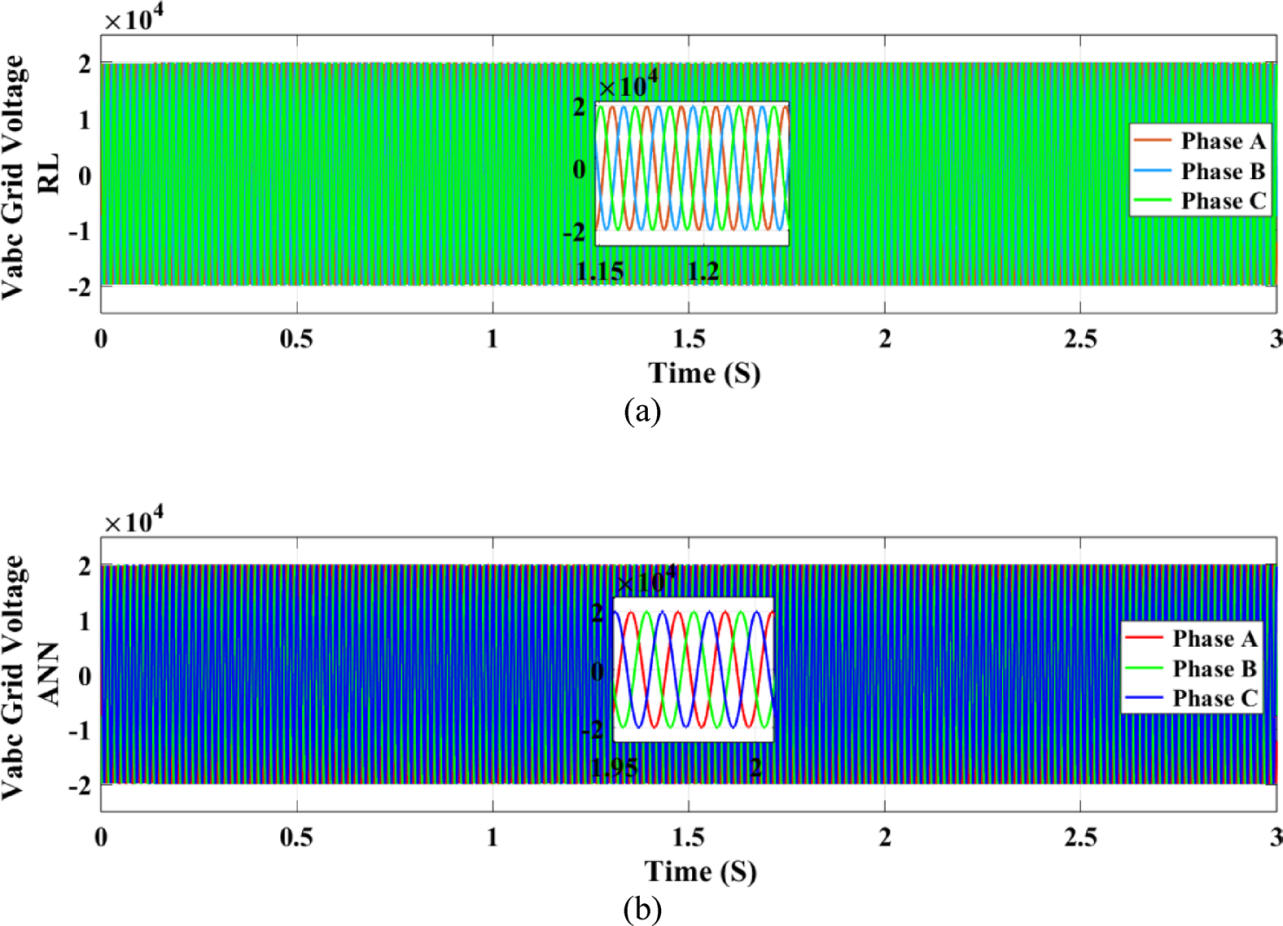
Grid voltage under random irradiance using both control systems (**a**) RL and (**b**) ANN.

The voltage stability for the RL and ANN control systems, respectively, is shown in Figs. 34(a) and 34(b). Stable voltage outputs are efficiently maintained by both systems. In contrast to the ANN system, the RL control system shows marginally smoother voltage regulation. This increased stability is important since stable voltage has a direct impact on the solar system’s power production and overall performance. These results demonstrate that although both control techniques work well, voltage stability is somewhat greater with the RL approach.

Table 4 shows the efficiency of the ANN and RL control techniques for a grid-connected PV system under various solar irradiance situations is contrasted in this table. According to the results, grid power (P_Grid_) varies between 23.86 kW and 33.99 kW, while PV power (P_PV_) for the ANN controller spans from − 6.15 kW to 41.78 kW. The RL controller, on the other hand, displays higher and more constant figures, with grid power falling between 40.45 kW and 56.43 kW and PV power between 49.29 kW and 57.93 kW. Furthermore, with RL, the PV voltage (V_PV_) continuously displays larger values, up to 286.72 V, as opposed to the maximum of 274.03 V under ANN. These results show that RL performs more consistently and effectively, particularly when exposed to different amounts of irradiance.

**Table 4:** Comparative of the main results for ANN and RL under random changes in solar radiation.

Controller	Irradiance (W/m^2^)	Time (s)	V_PV_ (V)	*P*_PV_ (kW)	*P*_Grid_ (kW)
ANN	455.25	0.15	238.71	41.78	33.99
	369.79	1.67	−42.01	−6.15	23.86
	607.89	2.34	274.03	57.93	56.43
RL	455.25	0.15	277.69	53.50	49.29
	369.79	1.67	286.72	41.94	40.45
	607.89	2.34	285.58	68.05	66.47

**Fig. 35:**
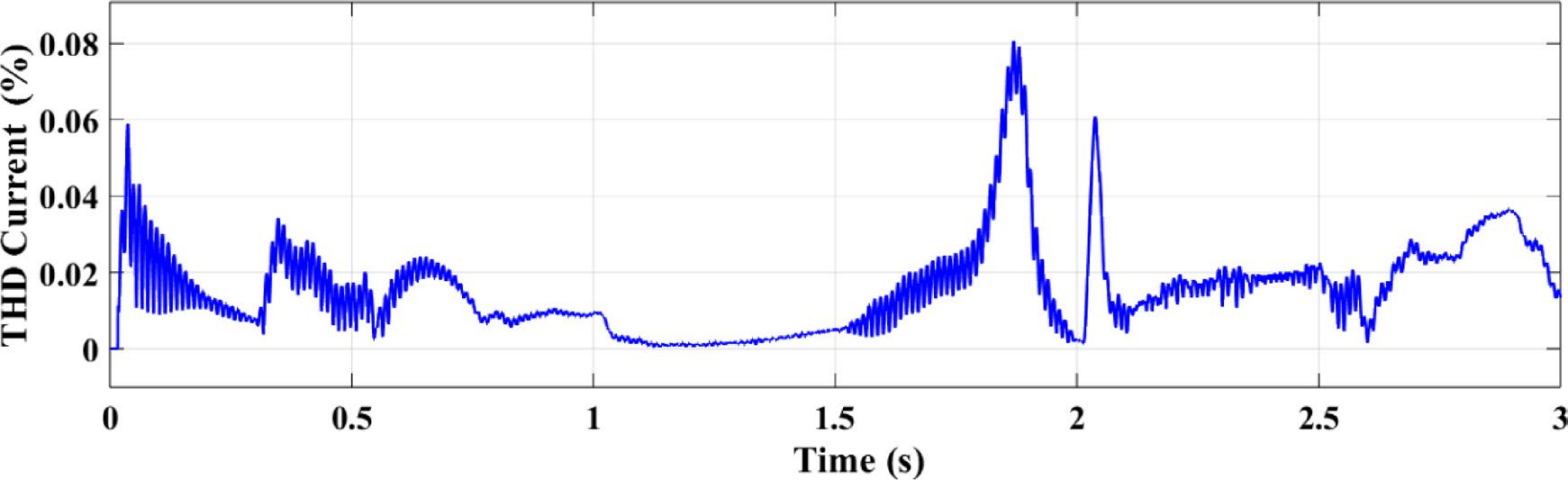
THD of the current using ANN under random conditions.

**Fig. 36:**
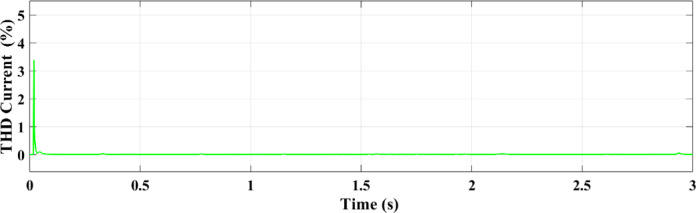
THD of the current using RL under random conditions.

Using the ANN controller, Fig. 35 shows the current THD under random solar radiation. The greater performance of RL in handling dynamic situations is demonstrated by the decreased harmonic distortions in the THD of the current under ramp conditions when utilizing the RL controller as opposed to the ANN controller as shown in Fig. 36.

**Fig. 37:**
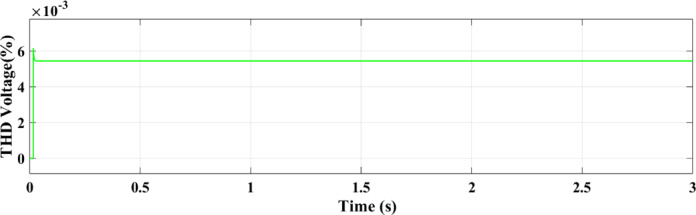
Voltage THD with ANN.

**Fig. 38:**
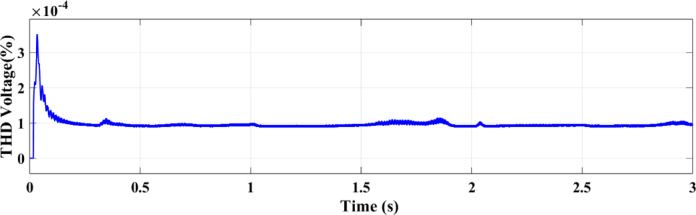
Voltage THD with RL.

Using the ANN controller, Fig. 37 displays the THD of the voltage under random solar radiation. The RL controller’s THD for voltage under ramp settings, which exhibits improved voltage regulation and less distortions as seen in Fig. 38, validates its ability to adapt to changing environmental conditions.

## Conclusion and future work

### Conclusion

This paper introduced two different controllers depending on AI techniques, ANN compared with RL with PV micro-grid connected double stage system. There are two types of differences between the two controllers, the first is a difference in the waveforms and the second is a difference in the value. From those results in all output figures, the results show the superiority of the modern technology controller RL in everything. The digital results from the PV microgrid system reveal significant differences between the RL and ANN controllers. The RL controller achieves an active power output. of 100 kW, while the ANN controller outputs 82 kW. This indicates that the RL approach, an advanced AI technique, increases power by 20% compared to the ANN controller. Additionally, the output current from the PV to the microgrid is 6 amps with the RL controller versus 4 amps with the ANN controller, representing a 2-amp increase with RL. The RL AI technique demonstrates superior performance compared to the ANN technology across various metrics. The comparison of the ANN and RL controllers shows that RL offers more robust and consistent performance in dynamic conditions in addition to increasing the total power output. ANN performs well, particularly under steady settings, although its accuracy declines by temperature or sun irradiance variations. The PV microgrid system functions successfully in a variety of circumstances because to the RL controller’s superior ability to adjust to these changes. By continually learning from its surroundings, RL can maximize the system’s performance in real-time, which emphasizes its major advantage over more conventional AI techniques like ANN. The enhanced adaptability and performance of RL demonstrate its potential to maximize the efficiency of renewable energy systems, making it a more suitable choice for modern PV microgrid applications. THD for both current and voltage under ramp and random circumstances is a summary of how well ANN and RL controllers perform in comparison. In both dynamic and uncertain contexts, the RL controller performs better because it consistently produces reduced THD values.

### Future work

Future research could focus on implementing the RL controller in real-time hardware platforms such as DSPACE to validate its performance under practical conditions. In addition, exploring hybrid AI models and deep reinforcement learning (DRL) techniques may help improve control accuracy while reducing computational complexity. It is also recommended to extend this approach to hybrid renewable systems and incorporate predictive maintenance and fault detection to enhance system reliability and operational flexibility.

## Data Availability

All data generated or analyzed during this study are included in this published article.
